# Assessing Inhalation Exposures Associated with Contamination Events in Water Distribution Systems

**DOI:** 10.1371/journal.pone.0168051

**Published:** 2016-12-08

**Authors:** Michael J. Davis, Robert Janke, Thomas N. Taxon

**Affiliations:** 1 Argonne Associate of Seville, Environmental Science Division, Argonne National Laboratory, Argonne, Illinois, United States of America; 2 National Homeland Security Research Center, U.S. Environmental Protection Agency, Cincinnati, Ohio, United States of America; 3 Global Security Sciences Division, Argonne National Laboratory, Argonne, Illinois, United States of America; Purdue University, UNITED STATES

## Abstract

When a water distribution system (WDS) is contaminated, short-term inhalation exposures to airborne contaminants could occur as the result of domestic water use. The most important domestic sources of such exposures are likely to be showering and the use of aerosol-producing humidifiers, i.e., ultrasonic and impeller (cool-mist) units. A framework is presented for assessing the potential effects of short-term, system-wide inhalation exposures that could result from such activities during a contamination event. This framework utilizes available statistical models for showering frequency and duration, available exposure models for showering and humidifier use, and experimental results on both aerosol generation and the volatilization of chemicals during showering. New models for the times when showering occurs are developed using time-use data for the United States. Given a lack of similar models for how humidifiers are used, or the information needed to develop them, an analysis of the sensitivity of results to assumptions concerning humidifier use is presented. The framework is applied using network models for three actual WDSs. Simple models are developed for estimating upper bounds on the potential effects of system-wide inhalation exposures associated with showering and humidifier use. From a system-wide, population perspective, showering could result in significant inhalation doses of volatile chemical contaminants, and humidifier use could result in significant inhalation doses of microbial contaminants during a contamination event. From a system-wide perspective, showering is unlikely to be associated with significant doses of microbial contaminants. Given the potential importance of humidifiers as a source of airborne contaminants during a contamination event, an improved understanding of the nature of humidifier use is warranted.

## Introduction

Drinking water distribution systems (WDSs) can be contaminated either intentionally or accidentally. Such contamination has the potential to cause adverse health effects in the population using water from the system and a number of studies have considered the consequences of ingesting water from a contaminated distribution system [1–9]. The potential also exists for short-term inhalation exposures to airborne contaminants during a contamination event. Various domestic uses of water can release volatile contaminants, generate contaminated aerosols, or do both. The largest inhalation exposures to volatile contaminants in water result from showering [10]. A screening-level assessment found that ultrasonic and impeller (cool-mist) humidifiers and showering are the largest sources of inhalation doses of aerosol-borne contaminants generated by common water uses [11]. Unlike ingestion exposures, potential inhalation exposures during a contamination event in a WDS have received little attention. One study assessed the potential for acute health consequences from the inhalation of aerosols that contain naturally occurring endotoxin from drinking water and found a possibility of acute responses associated with the use of aerosolizing humidifiers [12]. A second study considered the possible inhalation of contaminated aerosols during showering and suggested that the pathway may be relevant for highly infectious pathogens [9]. Drinking water may contain opportunistic pathogens that are present in household plumbing [13]. However, the consideration of any adverse effects resulting from the inhalation exposure to such pathogens is outside the scope of this paper, which considers only contamination that is present in the distribution system.

Considerable effort has been devoted to studying the volatilization of contaminants from drinking water, in particular from showering [10, 14–30]. Aerosol production associated with showering [30–32] and the use of portable humidifiers [33–38] has also been examined. The purpose of these studies generally has been to assess chronic exposures to contaminants, in particular disinfection byproducts present in the water. A detailed examination of chronic inhalation exposures to contaminated drinking water, emphasizing volatile contaminants, has been provided in a monograph edited by Olin [39].

This paper presents a simulation-based approach for assessing short-term, water-distribution-system-wide inhalation exposures that could result from showering and the use of humidifiers during contamination events. Assessing these exposures requires estimating contaminant concentrations in water at points of water use throughout the distribution system during the contamination event, accounting for the behavior of simulated individuals who use water from the system, estimating air concentrations of contaminants at points of exposure at the time when those individuals are using water, estimating inhalation doses for the individuals, and finally determining statistics for system-wide results. An application of the approach is presented using models for a number of actual water distribution systems. To provide some context for the results related to inhalation exposures, potential ingestion exposures associated with drinking contaminated tap water also are considered briefly.

The framework presented here can be applied in a variety of ways. The approach has been incorporated into U.S. EPA’s TEVA-SPOT software [40], which allows users to carry out consequence assessments for contamination events and to design contamination warning systems to help determine if such events have occurred. The capabilities provided for addressing potential inhalation exposures complement those already developed for addressing ingestion of contaminated tap water [8]. The framework is useful for applications involving both intentional and inadvertent contamination of a drinking water distribution system. Examples of both types of events are discussed by Janke et al. [41]. The framework can be used for assessing the magnitude of potential consequences, examining the relative significance of different potential exposure pathways, and prioritizing areas with the highest potential for problems. With a sufficiently accurate or real-time network model of the drinking water distribution system, the framework presented could be used to support the determination of the location or locations at which a contaminant was introduced and help determine the population of water users most likely to be affected. A bounding analysis presented in this paper may also be useful for carrying out screening evaluations of different WDSs for policy purposes. Only limited information is needed to perform the bounding analysis; a WDS network model is not required. In addition to researchers, potential users of the methods presented here include water utility managers or operators, consultants, and first responders.

The next section presents the assessment approach, which builds on considerable relevant past effort but required development of new methods for modeling the behavior of potentially exposed individuals and the integration of the various elements of the approach to provide system-wide results. Section 3 presents an application to three water distribution systems. A discussion of the results of the application and issues related to our approach are given in Section 4. Finally, Section 5 presents a summary and the conclusions of the paper.

## Methods

Determination of the adverse health effects associated with a contamination event in a WDS requires estimating the quantity of contaminant that is inhaled or ingested by individuals who use water from the system. Obtaining such estimates requires determining the air and water concentrations of the contaminant at the locations where exposures occur and accounting for the behavior of the individuals at those locations. Table 1 summarizes the various steps involved in determining inhalation and ingestion doses for a simulated individual. Approaches for estimating water concentration and ingestion doses are available [8]. The focus here is on estimating air concentrations of a contaminant at the point of exposure and accounting for the behavior of simulated individuals that results in exposure to airborne contaminants. Showering and use of humidifiers are considered separately.

**Table 1:** Estimation of Doses.

Step	Approach	Reference
Simulate contaminant injection	Independent injection at each NZD node	[8]
Simulate transport in WDS	Conservative tracer using EPANET [42]	[8]
Calculate air concentration	Mass balance, aerosols	[32]
	Mass balance, volatiles	[30, 43]
Calculate ingestion dose	Probabilistic timing model, ingestion	[8]
	Probabilistic volume model, ingestion	[8]
	Dose model	[8]
Calculate inhalation dose	Probabilistic timing model, showering	This paper
	Probabilistic duration model, showering	[44]
	Probabilistic frequency model, showering	[44]
	Timing and duration models, humidifier	This paper
	Dose model for showering: aerosols	This paper
	Dose model for showering: volatiles	This paper
	Dose model for humidifier: aerosols	This paper

WDS, water distribution system; NZD, nonzero demand.

Our interest is in system-wide effects. Therefore, the estimated doses for all simulated individuals using water from a WDS must be determined for the period of interest and analyzed collectively. The measure of adverse effects associated with a contamination event that we use, which is called **impact** in this paper, is the size of the population that receives a contaminant dose above some specified level by either inhalation or ingestion of contaminants from drinking water.

All simulations done for this paper used the latest version of U.S. EPA’s TEVA-SPOT software [40]; the approach presented here has been incorporated into its Health Impacts Assessment Module, part of the tool’s Consequence Assessment Module [45]. TEVA-SPOT uses EPANET [42] to simulate contaminant transport in a water distribution system. EPANET is the standard software for hydraulic and water quality simulations in distribution systems. WDSs are represented by network models that are used as input to EPANET.

The following subsections provide the details of our approach. First, the methods used to account for contaminant injection into a WDS and for contaminant transport in the distribution system are presented briefly. The behavior models for showering, the models for estimating inhalation doses associated with showering, and the determination of parameter values for these models are considered next. Behavior models for humidifier use, models for estimating inhalation doses associated with humidifier use, and the determination of parameter values for these models are then presented. Finally, determination of system-wide consequences and the analysis of such consequences are discussed. The method used to estimate ingestion doses has been described previously [8] and the details are not repeated here. All simulated individuals are assumed to have the potential to ingest contaminated water. Probabilistic models were used to describe the times when ingestion occurs and the volumes of water ingested.

### Contaminant injection and transport

We assumed that contaminants can be injected at any node (pipe junction) in a WDS with a nonzero demand (NZD) and carried out independent simulations for the entire network for each such location. For example, for a network with N NZD nodes, there would be N independent simulations for each application being studied. Each of the N simulations is called a scenario and the collection of scenarios for a network is called an ensemble. We assume an injection of 10 kg of a contaminant at 0:00 hours local time. Assuming a concentration of 10^14^ cells/kg, this is equivalent to an injection of 10^15^ cells for cases involving microbial contaminants.

Use of 10 kg of a microbial contaminant with a concentration of 10^14^ cells/kg is feasible. A high level of purity is not necessary. Concentrations of up to 10^14^ to 10^15^ living bacterial cells/kg occur in the luminal contents of the large intestine [46]. For bacteria with a mass of 10^-12^ g/cell and that are 67% water, a concentration of 10^14^ cells/L corresponds to a cell dry weight of 33 g/L. In applications in which bacteria are used to produce some product, if the mass of the product is 75% of the cell dry weight, the titer (mass of product/L) would be 25 g/L for such a cell dry weight. Microbial production of chemicals and materials at pilot and industrial scales can yield cell dry weights/titers greater than these amounts [47–51]. (A titer over 50 g/L could be the minimum necessary for production at an industrial scale [52].) This is an area of active interest and improvements and expansion to new products are expected [52–54].

As discussed below, the estimates of impacts presented in this paper can be scaled to accommodate injection quantities different from the ones used here. The injection quantities used should only be considered as feasible or possible.

The approach presented here can accommodate a wide variety of possible contaminants, including those that behave conservatively (i.e., persist) in the distribution system as well as those that decay during transport. This paper considers only conservative behavior, which provides a conservative estimate of consequences.

In our simulations, TEVA-SPOT used EPANET version 2.00.12 (with hydraulic and reporting time steps both equal to 1 h and a water-quality time step equal to 1 min) to determine contaminant concentrations at all network nodes downstream from the injection node. All simulations were one week (168 h) in duration. Although this paper considers injections at all NZD nodes, the framework presented here can be applied to injections at any subset of nodes in a network.

After contaminant concentrations were determined at downstream nodes, inhalation and ingestion doses were determined for all simulated individuals using water from those nodes. Any influence of household plumbing on the timing of contaminant pulses at the exposure location was neglected. Network population was assumed to be distributed across network nodes in proportion to average nodal water demands. The average demand at a node is the average over all the demand patterns for the node, with each pattern repeated to the least common multiple of the lengths of all the patterns. In an EPANET-based network model, a demand pattern characterizes the time variation in water usage (demand) at a node.

### Showering: Behavior models

Determining inhalation doses associated with showering requires knowing who showers, when they shower, and the duration of the showering. Contaminant concentration in the water supplied to a shower changes with time during a contamination event. Therefore, knowledge of timing is important. The frequency with which people shower and the duration of showers have been quantified [44]. A recent study presented a simple timing model for showering using data collected in the Netherlands [9]. The model assumes that individuals take at most one shower per day and that times for showering can be described by a mixture of two normal distributions with peaks in the morning and evening; however, no details were provided on the data used to develop the model. We are not aware of any other or any more detailed models for the timing of showers and, therefore, include the development of such a model here.

Given the limitations in available data, a number of assumptions are necessary when developing a model that includes showering frequency, duration, and timing. In particular, the major assumptions are the following: (1) frequency, duration, and timing of showering are independent; (2) the behavior of a simulated individual is the same for each day in a simulation; and (3) the timing of grooming events as reported in time-use studies serves as an adequate proxy for the timing of showering events. We did not attempt to relate showering behavior to an individual’s characteristics (e.g., age, sex).

#### Frequency and duration

Showering frequency can be determined using results for the U.S. presented in Table II of Wilkes et al. [44]. Considering individuals of all ages, 22% do not take a shower, 60% take one shower each day, 17% take two showers per day, and less than 1% take three or more showers per day.

Showering duration can be determined using results for the U.S. presented in Table V or Fig 4 of Wilkes et al. [44] for the Residential End Uses of Water Study data set. Results for duration in this data set are based on actual measured water use obtained from a household water meter. The distribution of showering durations is approximately lognormal. The parameters for the distribution are *μ* = 1.92 and *σ* = 0.493. These quantities are given on a logarithmic scale. (Given in minutes, they are exp(1.92) = 6.8 and exp(0.493) = 1.64.) Mean shower duration is 7.70 min.

After a shower is turned off, exposure to airborne contaminants continues until an individual leaves the shower stall. However, no results appear to be available for the time an individual spends in the shower stall after the shower is turned off; we have no data for this quantity. We assume that the value is a constant and equal to two minutes and consider the sensitivity of results to this assumption below.

#### Timing

Estimates for the times when showering occurs were developed using data for the U.S. collected from 2003 to 2012 by the American Time Use Survey (ATUS) [55]. ATUS is a continuous survey on time use that has been sponsored by the Bureau of Labor Statistics and conducted by the U.S Census Bureau since 2003. Its primary purpose is the development of nationally representative estimates of how Americans spend their time. The universe for ATUS sampling is the civilian, non-institutional population living in occupied households who are at least 15 years of age. ATUS uses a stratified, three-stage sample that is obtained from a subset of households interviewed by the Current Population Survey, a monthly survey of about 60,000 households, which is the primary source of statistics on the U.S. labor force. About 40,500 households were included in ATUS in 2003 and about 26,400 have been included in subsequent years. Annual response rates have varied from about 53 to about 58%. Data are collected from a randomly selected, eligible person from each household using computer-assisted telephone interviewing. Persons designated for inclusion in the survey are notified in advance and given information on the survey. Respondents provide time-use information for the 24-h period that starts at 4 a.m. on the day prior to the interview and ends on 4 a.m. on the day of the interview.

**Fig 1:**
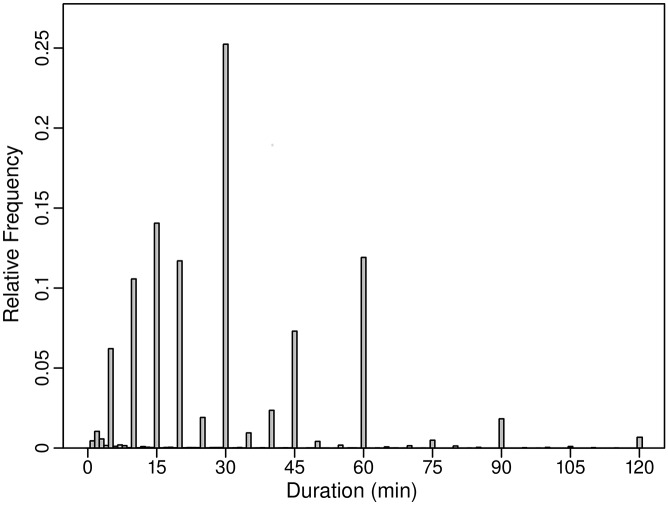
Weighted histogram of durations of grooming events reported in ATUS from 2003 to 2012. Only events with a duration ≤ 120 min are included. N = 178,152.

The ATUS category labeled grooming (Category 010201 in the classification scheme used) includes showering. Grooming was used as a proxy for showering and the starting time for a grooming event was used as the starting time for a showering event. Grooming includes activities other than showering. The reported durations of grooming events vary substantially. There were 178,676 grooming events reported for the 10-year period 2003 through 2012. Of these, 524 exceeded 120 minutes in length, while 34,395 were less than 15 minutes in length. Fig 1 shows the weighted distribution of durations for events that are 120 min or less in length. Note in Fig 1 that respondents generally reported durations with lengths that are multiples of 5 min. About 87% of events have reported durations of 5, 10, 15, 20, 30, 45, or 60 min. Sampling weights for ATUS respondents can vary substantially. The ratio of the maximum to minimum weights in the sample is about 251. Consequently, consideration of the weights is important.

There is no way to determine which of the reported events included showering. However, duration is not an important influence on the starting time of an event. Considering all reported grooming events, starting time has only a small negative correlation with duration. The adjusted R^2^ is 0.036. Less than 4% of the variation in starting time is explained by duration. Consequently, although showering did not occur in all grooming events and the occurrence of showering may be correlated with event duration (e.g., some events may be too short to accommodate showering), the starting times for events that include showering likely have a distribution similar to that for all grooming events. Therefore, the distribution of starting times for events that include showering is assumed to be the same as the distribution of starting times for grooming events. It is unlikely that showering begins immediately at the start of a grooming event. However, there is no information available on the delay between the start of a grooming event and the start of a showering event. Therefore, the delay is assumed to be zero.

**Fig 2:**
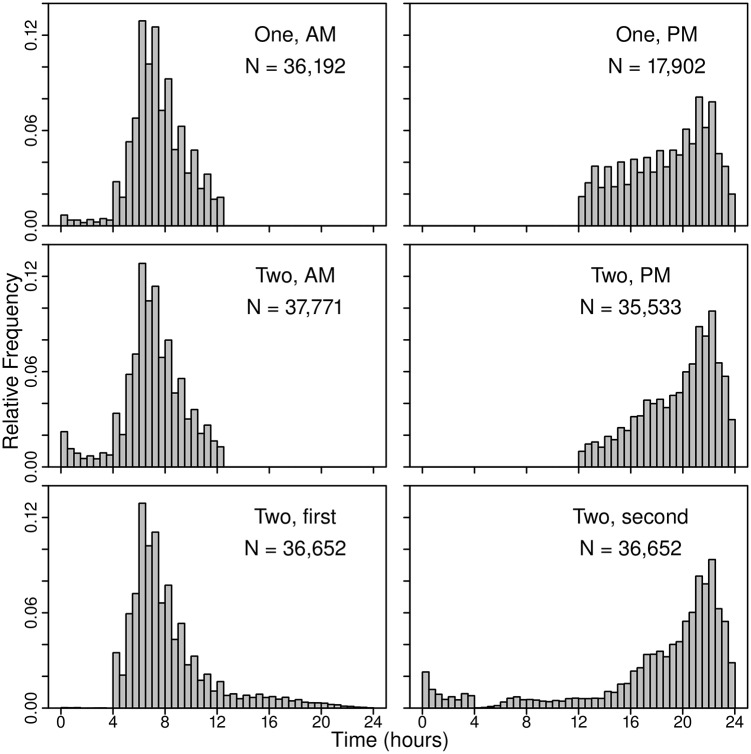
Weighted histograms of starting times of grooming events reported in ATUS from 2003 to 2012. Results are presented for cases for which one and two events were reported. Results for single events are separated into cases in which events occurred before noon and after noon (top row). For cases with two events, results are presented separately for events occurring before and after noon (middle row) and also for the first and second of the two events (bottom row).

As noted above, less than 1% of the U.S. population takes more than two showers per day. Therefore, we neglect cases in which individuals take more than two showers per day and consider only cases in which individuals take no showers or one or two showers per day. There is no basis for relating the number of reported grooming events to the number of showers that an individual takes. However, there is likely to be a significant correlation between the two quantities. For example, consistency requires that an individual who reports one grooming event per day can have no more than one showering event per day. We assume that the starting times for showering events for individuals who take one shower per day are distributed in the same manner as the starting times for grooming events for individuals who report one grooming event per day. We also assume that the starting times for showering events for individuals who take two showers per day are distributed in the same manner as the starting times for grooming events for individuals who report two grooming events per day. Fig 2 shows the distribution of starting times for grooming events for individuals reporting one and two events. The distributions are separated into two parts. For cases with one event, distributions are shown for events reported in the morning and for events reported after noon. For cases involving two daily events, distributions also are shown for the events reported for the morning and for events reported to take place after noon. In addition, for cases involving two events, distributions are shown for the first of the two events and for the second of the two events. Note the similarity of the distributions for starting times for morning and afternoon events for cases involving one event to the distributions for starting times for morning and afternoon events for cases with two events.

**Fig 3:**
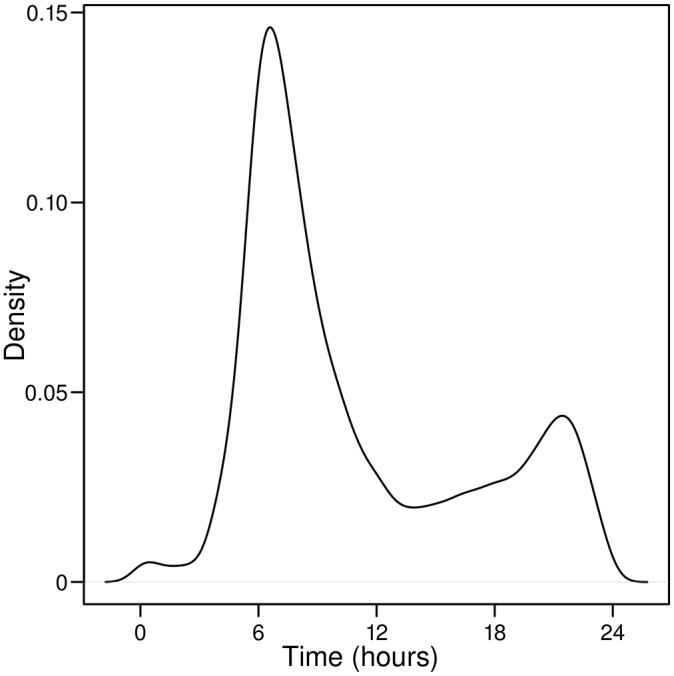
Estimated weighted probability density function for starting times of single ATUS grooming events. N = 54,094.

**Fig 4:**
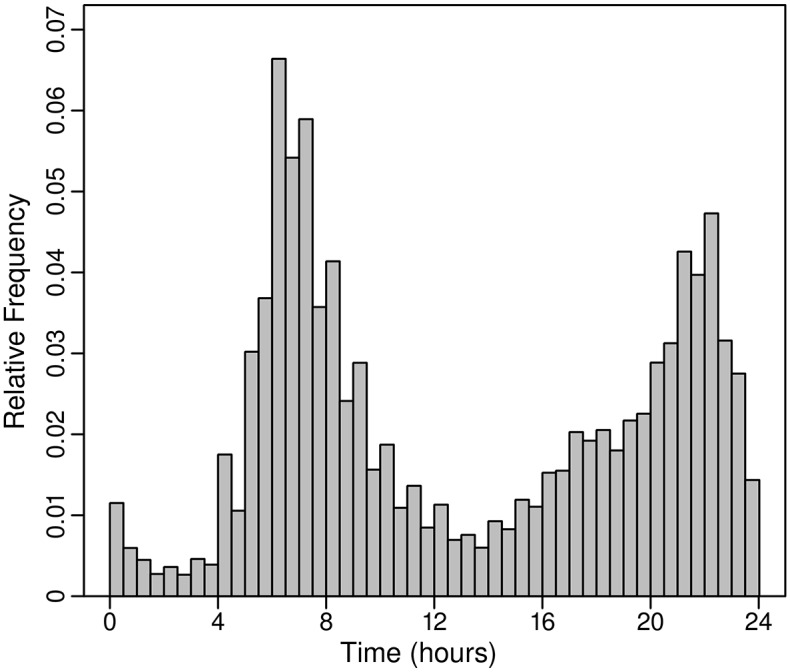
Weighted histogram of starting times for cases with two ATUS grooming events. N = 36,652.

In our simulations, starting times for simulated individuals taking one shower per day were obtained using an empirical probability distribution based on ATUS timing data for grooming. The estimated probability density function for starting time is shown in Fig 3 for ATUS data for grooming events for the period 2003 through 2012 for individuals who reported a single grooming event. The estimate is based on results from a weighted sample of 54,094 individuals. When a second shower is taken, its timing likely is influenced by the time of the first shower. In our simulations for individuals taking two showers per day, we avoided issues related to trying to account for this dependency using probability distributions by using random samples of actual starting times for ATUS respondents who reported two grooming events. For example, if in a simulation there were 1,000 individuals who take two showers per day, we randomly selected, with replacement, 1,000 cases from the ATUS results in which an individual reported two grooming events and used the two starting times for each case for one of the 1,000 individuals in the simulation. Fig 4 shows the histogram for starting times for individuals reporting two events, again for the period 2003 through 2012. The histogram is based on weighted results for 36,652 individuals reporting two events. Starting times, shower frequency, and shower duration for an individual are assumed to remain the same for all days in a simulation.

Starting times for grooming events vary somewhat by day of the week. For individuals reporting a single grooming event per day, events reported to occur in the morning on weekend days tend to take place later than those reported for week days and more events are shifted to later in the day on weekend days than on week days. For individuals reporting two grooming events per day, starting times for the first event tend to be later for weekend days than week days; for the second event, starting times on Fridays and Saturdays tend to be shifted to earlier in the day than for the other days. When all days are considered, the starting times for cases involving one or two events per day are similar to those for weekdays. We used results for all days together and did not consider any variations in starting time that occur with day of the week. We generally do not have any information on how the various quantities that we need to consider (e.g., showering duration, water distribution system hydraulics) vary with day of the week. Therefore, we have neglected any influence such variation may have.

### Showering: Estimating inhalation doses

Estimating the inhalation dose associated with showering requires determining the air concentration of the contaminant in the shower stall during a shower. Contaminants may be present in shower air as a gas or contained in aerosols.

Using models for the physical processes affecting the air concentration of a contaminant in a shower, concentrations were determined for the shower stall. These concentrations, together with an average breathing rate, were integrated over the duration of the shower to obtain the estimates for inhalation doses presented here. These doses are the mass of the contaminant that enters the body by inhalation and are actually potential inhalation doses because not all of the contaminant mass will necessarily remain in the body. Some contaminant mass can be exhaled before being absorbed. Doses need to be determined separately for each showering event for each simulated individual because contaminant concentration varies with time during a simulation. Doses for each of the separate showering events for each individual need to be added to obtain a cumulative dose for each individual for the period of the simulation.

**Fig 5:**
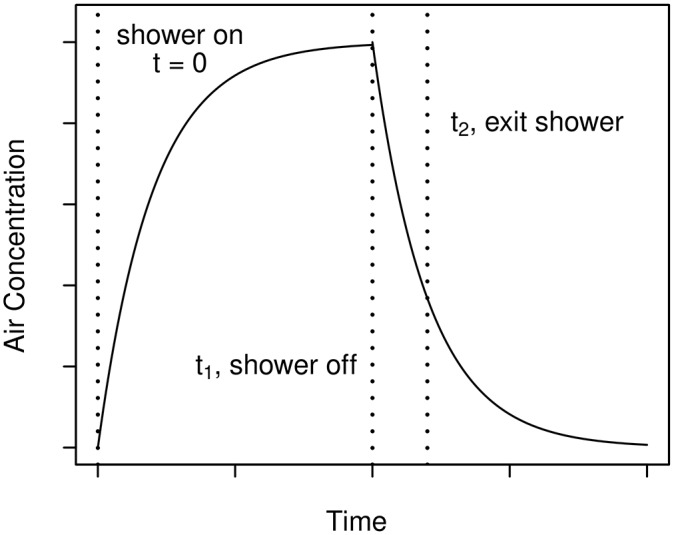
Behavior of contaminant concentration in air in a shower stall.

Contaminant concentration in the air in a shower stall during a shower varies approximately as shown in Fig 5. The concentration starts at zero, increases with time, and, if the shower continues long enough, reaches an equilibrium, assuming that the contaminant concentration in the water being used does not vary during the course of a shower. At some time *t*_1_ the shower is turned off and the air concentration begins to decrease. The individual taking the shower exits the shower at time *t*_2_. The air concentration as a function of time can be estimated using a mass-balance approach for the shower. Air concentration is estimated separately for the period from *t* = 0 to *t* = *t*_1_ and the period from *t* = *t*_1_ to *t* = *t*_2_. Air concentration of contaminant has a similar variation with time for both volatile contaminants and for contaminants that are present on aerosol particles in the shower. However, the physical processes involved are different and separate calculations are required.

The air concentration of the contaminant (*C*_*a*_, in mg/m^3^ or cells/m^3^) can be determined using the following transient mass-balance equation:

$$\begin{array}{c} V_{s}\frac{dC_{a}}{dt}=\text{generation}\;\text{rate}\;‐\;\text{loss}\;\text{rate} \end{array}$$ (1)

where *V*_*s*_ is the shower stall volume (in m^3^) and the generation and loss rates are the rates at which the contaminant is being generated in or lost from the air in the shower stall (in mg/min or cells/min). In all cases considered here, instantaneous and complete mixing of contaminant in the shower stall is assumed.

#### Contaminants in aerosols

Assuming no partitioning of the contaminant when aerosols are produced, the rate at which contaminant mass associated with aerosols is generated during showering is *fG*_1_, where *f* is the mass fraction (in mg/mg) or number fraction (in cells/mg) of the contaminant in the water and *G*_1_ (in mg/min) is the rate at which aerosol mass is generated. *G*_1_ must be determined empirically. The loss rate is *λ*_*on*_ *C*_*a*_ *V*_*s*_, where *λ*_*on*_ (in min^−1)^ is the removal rate for aerosols while the shower is on. The removal rate equals the sum of the air exchange rate for the shower stall and the loss rate of particles due to deposition, which depends on particle size. If the air concentration of a contaminant in aerosols is zero at *t* = 0, the air concentration as a function of time is then [32]

$$\begin{array}{c} C_{a}\left(t\right)=\frac{fG_{1}}{\lambda_{on}V_{s}}\left(1-exp\left(-\lambda_{on}t\right)\right) \end{array}$$ (2)

When the shower is turned off at time *t*_1_, the generation rate becomes zero and the solution of Eq (1) is

$$\begin{array}{c} C_{a}\left(t\right)=C_{a}\left(t_{1}\right)exp\left(-\lambda_{off}\left(t-t_{1}\right)\right) \end{array}$$ (3)

for *t* > *t*_1_, where *λ*_*off*_ (in min^−1^) is the removal rate after the shower is turned off.

The time-varying concentration of the contaminant in the air, along with an average breathing rate, was integrated to give the inhalation dose, *D*, (mass in mg for chemical contaminants or number of cells for microbial contaminants) for one shower event. For contaminants in aerosols, considering both the time when the shower is on (Eq (2)) and the time after it is turned off (Eq (3)), the inhalation dose is the following:

$$\begin{array}{ccc} D & = & \frac{BfG_{1}}{\lambda_{on}V_{s}}(t_{1}-\frac{1}{\lambda_{on}}\left(1-exp\left(-\lambda_{on}t_{1}\right)\right)+\\ & & \frac{1}{\lambda_{off}}\left(1-exp\left(-\lambda_{on}t_{1}\right)\right)\left(1-exp\left(-\lambda_{off}\left(t_{2}-t_{1}\right)\right)\right)) \end{array}$$ (4)

where *B* is the breathing rate in m^3^/min.

The duration of a shower, *t*_1_, was determined randomly for each simulated individual taking a shower using the lognormal distribution for showering durations discussed above. Lacking any data, as noted above, it was assumed that the time a person remains in the shower stall after the shower is turned off, *t*_2_ − *t*_1_, is a constant value.

The value for *f* is obtained from simulation results for the contaminant concentration in water at the exposure location. Again, values for *G*_1_ must be determined empirically. A review of available values is provided below. Values for other parameters in Eq (4) are also discussed below.

#### Volatile contaminants

Air concentrations of volatile contaminants in a shower stall can be estimated in different ways. One approach is to account for contaminant movement from shower water to air using a factor that represents the efficiency with which contaminants are transferred from water to air. Another is to account for this process using mass-transfer coefficients. An objection to the use of transfer efficiency is that a value applies only to a specific substance [43]. If a mass-transfer coefficient is available for some compound for a particular process (e.g., showering), it can be adjusted to provide an estimate for a mass-transfer coefficient for a second compound [43]. However, mass-transfer coefficients also can depend on flow rates and temperature, so they may not always be straightforward to use. When we consider actual WDSs, we use a model to estimate air concentrations of contaminants based on transfer efficiencies. We are not interested in any specific contaminants, but want to assess the potential significance of the inhalation of volatile contaminants during a contamination event. Transfer efficiency has an intuitive appeal and using a generic value allows us to evaluate the significance of a general class of substances. (Inhalation doses for volatile contaminants can be determined with TEVA-SPOT using an approach based on either transfer efficiency or mass-transfer coefficients, although only the first approach is used here.)

Using an approach for volatile contaminants based on a transfer-efficiency coefficient, (*T*, unitless), the generation rate, *G*_2_ (in mg/min) equals *C*_*w*_ *Q*_*w*_ *T*, where *C*_*w*_ is the contaminant concentration (in mg/m^3^) in the water entering the shower stall and *Q*_*w*_ (in m^3^/min) is the flow rate for water entering the shower stall. The loss rate for volatile contaminants is *C*_*a*_ *Q*_*a*_, where *Q*_*a*_ (in m^3^/min) is the rate at which air leaves the shower stall. The loss rate can also be expressed as *λ*_*aer*_ *C*_*a*_ *V*_*s*_, with a removal rate *λ*_*aer*_ = *Q*_*a*_/*V*_*s*_ = the air exchange rate. The air concentration of a volatile contaminant in the shower stall as a function of time when the shower is on is given by [30]

$$\begin{array}{c} C_{a}\left(t\right)=\frac{G_{2}}{\lambda_{aer}V_{s}}\left(1-exp\left(-\lambda_{aer}t\right)\right) \end{array}$$ (5)

It is assumed that the initial air concentration of the contaminant in the shower stall is zero and that the concentration of the contaminant in air entering the shower stall also is zero.

When the shower is turned off, contaminant concentration is given by an equation of the same form as that in Eq (3). However, the removal rate for volatiles is *λ*_*aer*_.

For volatile contaminants, the expression for inhaled dose is similar to the expression in Eq (4) for aerosols. The inhalation dose (in mg) for volatiles for one showering event obtained using the transfer-efficiency approach is the following:

$$\begin{array}{ccc} D & = & \frac{BG_{2}}{\lambda_{aer}V_{s}}(t_{1}-\frac{1}{\lambda_{aer}}\left(1-exp\left(-\lambda_{aer}t_{1}\right)\right)+\\ & & \frac{1}{\lambda_{aer}}\left(1-exp\left(-\lambda_{aer}t_{1}\right)\right)\left(1-exp\left(-\lambda_{aer}\left(t_{2}-t_{1}\right)\right)\right)) \end{array}$$ (6)

Times *t*_1_ and *t*_2_ have the same meaning for volatile contaminants as for contaminants in aerosols.

### Showering: Parameter values

Parameter values used in this study to estimate inhalation doses are summarized in Table 2. The basis for these values is discussed below.

**Table 2:** Parameters for Showering Models.

Process	Parameter	Description	Value
General	*B*	Breathing rate	0.012 m^3^/min
	*Q*_*w*_	Water flow rate	9 L/min
	*V*_*s*_	Shower stall volume	2 m^3^
Aerosolization	*G*_1_	Aerosol generation rate	6.0 mg/min
	*f*	Contaminant mass or number fraction	From simulation
	*λ*_*on*_	Removal rate, shower on	0.2 min^−1^
	*λ*_*off*_	Removal rate, shower off	0.1 min^−1^
Volatilization	*T*	Transfer-efficiency coefficient	0.8
	*λ*_*aer*_	Air exchange rate	0.2 min^−1^

#### General parameters

For breathing rate (*B*), the mean value for most age groups for light intensity activity, 0.012 m^3^/min, was used. This value is the recommended short-term exposure value for males and females combined [56].

We assumed a typical water flow rate (*Q*_*w*_) for a shower, 9 L/min. A shower stall volume (*V*_*s*_) of 2 m^3^ was used. This is the volume of a small shower and its use will make estimated doses more conservative (larger). We do not have any information on how other quantities, in particular shower duration, might be related to these two parameters. However, duration, for example, may possibly be correlated with them. Consequently, we did not attempt to use an independent distribution for the parameters.

#### Parameters for aerosolization

Table 3 summarizes results from studies that have reported aerosol mass generation rates for showers. Values for mass generation rate and removal rate are determined by fitting models similar to those described by Eqs (2) and (3) to measured aerosol particle mass concentrations.

**Table 3:** Aerosol Mass-generation Rate (*G*_1_, mg/min) and Removal Rates (*λ*, min^-1^) for Showers.

Study	*G*_1_ ^a^	*λ*_*on*_ ^a^	*λ*_*off*_ ^a^	Conditions^b^
Xu and Weisel [30]	3.6	1.24^c^ 0.35^d^	∼0.05^c^ ∼0.1^d^	36-36°C; *Q*_*w*_ = 7-10 L/min; *V*_*s*_ = 6.0 m^3^; N = 4 (> 2 μm), N = 6 (1-2 μm)^e^
Cowen and Ollison [31]	0.05^f^	0.18	0.11	15 and 38°C; *Q*_*w*_ ∼5 and ∼8 L/min; N = 19
Zhou et al. [32]	4.5^g^ 4.8^h^ 5.7^i^	0.20^g^ 0.32^h^ 0.40^i^	–	43-44°C; *V*_*s*_ = 2.0 m^3^; N = 1

^a^ Average values

^b^ Temperature is for water. *Q*_*w*_ is the water flow rate. *V*_*s*_ is the shower stall volume.

^c^ Particle diameter > 2 μm

^d^ Particle diameter in range 1-2 μm

^e^ Values of *G*_1_ and *λ* were determined for different aerosol size ranges; about 98% of mass was associated with aerosols with diameters greater than 1 μm.

^f^ Shower stall volume not given; assumed to be 3 m^3^ (a “full-size tub with shower curtain”) to estimate *G*_1_.

^g^ *Q*_*w*_ = 5.1 L/min.

^h^ *Q*_*w*_ = 6.6 L/min.

^i^ *Q*_*w*_ = 9.0 L/min.

Xu and Weisel [30] estimated mass generation and removal rates for eight aerosol size ranges extending from 0.1 to greater than 2.0 μm. The mass generation rate reported in Table 3 is the total for the overall size range. Removal rates are given in the table for the two largest size ranges, which contain about 98% of total aerosol mass. The average air exchange rates for the shower stall were about 0.14 and 0.06 min^-1^, when the shower was turned on and turned off, respectively. For aerosols with diameters less than 2 μm, the total removal rate while the shower was on was not significantly different from the air exchange rate. (The reported air exchange rate with the shower on was 0.14 ± 0.027 min^-1^ and the reported removal rate for aerosols with diameters in the range 1 to 2 μm, for example, was 0.35 ± 0.21 min^-1^.) For larger aerosols, processes other than convection and diffusion are also important. Aerosol concentrations were measured for a 6-min period before the shower was turned on, for a 10-min period while the shower was on, and for a 26-min period after the shower was turned off. After the shower was turned off, the removal rates for aerosols with diameters below 2 μm were about 0.1 min^-1^; larger aerosols had a significantly smaller removal rate, about 0.05 min^-1^, possibly due to being less affected by evaporation. Xu and Weisel [30] did not attempt any systematic analysis of the influence of water flow rate, water temperature, or air exchange rate on their results.

Cowen and Ollison [31] measured aerosol particle concentrations in six size ranges extending from 0.3 to greater than 10 μm. They estimated composite mass generation rates and decay rates. Measurements were made with a “full-size” mannequin in the shower stall. The average air exchange rate for the room containing the shower stall was about 0.01 min^-1^; no air exchange rate was reported for the shower stall. Particle concentrations were measured for a 5- to 10-min period before the shower was turned on, for a 10-min period with the shower on, and for a 10-min period after the shower was turned off. The average removal rate after the shower was turned off was 0.11 min^-1^, with minimum and maximum values of 0.04 and 0.18 min^-1^ for 19 runs. Cowen and Ollison [31] varied water temperature (∼15°C to ∼38°C), showerhead setting (coarse or fine), and flow rate (∼5 and ∼8 L/min) in their experiments. They found that no single variable was a major factor in determining estimated particle generation rate.

Zhou et al. [32] measured aerosol particle concentrations and mass concentrations of particles during showering and examined the effect of water temperature and flow rate. They estimated composite mass generation rates and decay rates. Measurements were made with a 1.7-m high mannequin in the shower stall. Three different showerheads were used, each with a different, constant flow rate (5.1, 6.6, and 9.5 L/min) and both hot (43-44°C) and cold (24-25°C) water. Results were presented for particle diameters less than about 30 μm. Table 3 summarizes only the results for measurements made using hot water. Measurements were made for “a few minutes” before the shower was turned on and for 10 min while the shower was on. The shower curtain was opened 2 min after the shower ended and bathroom doors were opened 5 min later. Measurements continued until room temperature and relative humidity returned to baseline values. The air exchange rates for the bathroom was 0.020 min^-1^ and 0.027 min^-1^ with bathroom doors closed and open, respectively. The particle size distribution did not vary while the shower was on. For cold water, the mass generation rate was only 0.06 to 0.17 mg/min, compared to a mass generation rate of 4.5 to 5.7 mg/min for hot water. Total removal rates for cold water were 0.66 to 0.77 min^-1^, considerably larger than for the hot water (0.20 to 0.40 min^-1^). Mass generation rate increased with flow rate for both hot and cold water. For hot water, flow rate had little effect on the particle size distribution; for cold water, it had a significant effect.

Xu and Weisel [30] and Zhou et al. [32] reported similar composite mass generation rates, but different removal rates for larger particles. Cowen and Ollison [31] reported a mass generation rate that is smaller by a factor of ten than that estimated in the other two studies, plus a smaller removal rate when the shower is on. Zhou et al. [32] suggested that the lower generation rate found by Cowen and Ollison [31] might be due to a larger air exchange rate and a lower water temperature. However, a larger air exchange rate seems inconsistent with a smaller removal rate. Zhou et al. [32] found that the aerosol mass generation rate increases with flow rate and that the mass generation rate is much higher for hot water than for cold water. However, they also found that the removal rate increases with flow rate for hot water. Estimated maximum equilibrium particle mass concentration, *G*_1_/*λ*_*on*_ *V*, is a maximum for the smallest flow they considered.

To obtain conservative results we assume hot water showers, a mass generation rate at the upper end of the range shown in Table 3 (6.0 mg/min), a relatively small removal rate (0.20 min^-1^, the lower end of the range in the table) when the shower is on, and a relatively large flow rate (9 L/min). We use a removal rate of 0.1 min^-1^ when the shower is off. This is the value in Table 3 reported for smaller diameter particles.

#### Parameters for volatilization

Table 4 provides values for the water-to-air transfer efficiency (*T*) for showers for a range of volatile chemicals. These results are not meant to be exhaustive, but to illustrate values of *T* for a range of substances. Transfer efficiencies for chemicals with a Henry’s law constant (dimensionless: $L_{liq}^{3}/L_{gas}^{3}$) greater than or equal to the value for toluene should vary from 0.6 to 0.8 [26]. In other words, highly volatile chemicals will have values for *T* in this range.

**Table 4:** Selected Transfer Efficiencies (*T*) for Volatile Chemicals in Showers.

Substance	*T* ^a^	Conditions and Reference^b^
Radon	∼0.60 and ∼0.70	22°C; custom shower head; *Q*_*w*_ = 2 or 4 L/min; N not given [24]
Seven VOCs^c^	0.59 to 0.75 × *T*_*Rn*_	Estimated using value for radon [16]
Five VOCs^d^	0.11 to 0.73	21 to 36°C; adjustable low-flow shower head; *Q*_*w*_ = 6.1 or 9.1 L/min; N = 10 [26]
DCP and TCP^e^	0.17 and 0.11	36-38°C; domestic showerhead; *Q*_*w*_ ∼8-9 L/min; N = 20 [30]
Chloroform	0.65	40°C; standard domestic water-saving nozzle; *Q*_*w*_ not specified, adjusted to obtain desired water concentration of contaminant; N = 18 [25]
TCE^f^	0.61	22 or 37°C; nozzle not specified; *Q*_*w*_ = 9.5 L/min; N = 42 [19]
TCE	0.87	40-50°C; domestic water-saving nozzle; *Q*_*w*_ = 6 L/min; N = 6 [23]

^a^ Average measured values except as noted. *T*_*Rn*_ is the value for radon.

^b^ Temperature is for water. *Q*_*w*_ is the water flow rate.

^c^ Carbon tetrachloride, chloroform, ethylene dibromide, dibromochloropropane, 1,1,1-trichloroethane, tetrachloroethylene, and trichloroethylene.

^d^ Acetone, ethyl acetate, toluene, ethylbenzene, and cyclohexane.

^e^ DCP: 1,1-dichloropropanone; TCP: 1,1,1-trichloropropanone.

^f^ TCE: trichloroethylene.

Transfer efficiency can depend not only on the compound but also on the specific details associated with the showering event. Differences in the design of the shower nozzle can influence the transfer efficiency [25], but how domestic shower nozzles are adjusted (i.e., spray vs jet; coarse vs fine) seems to have little effect [25, 26]. Water temperature has been reported to have no significant influence on transfer efficiency [19], to have an influence for some nozzles [25], and to be the major influence [26]. A study that considered water flow rates of 6.1 and 9.1 L/min found that flow rate had little effect on transfer efficiency [26]. Transfer efficiency can change during the course of a showering event, decreasing with time as the air concentration of the contaminant increases [26]. For highly volatile chemicals (e.g., toluene), transfer efficiency in a shower stall stabilizes rapidly, but for low-volatility chemicals (e.g., methyl ethyl ketone), transfer efficiency may not stabilize for some minutes [26].

Methods exist for estimating transfer efficiencies for compounds for which measurements are not available [16, 26]. Values for water and air diffusion coefficients and Henry’s law constant are needed for the compound of interest and also for a reference compound (e.g., radon), for which appropriate measurements are available.

To provide conservative illustrative results for a generic compound in our assessment, we used a value for *T* equal to 0.8, as shown in Table 2, from the upper range of values given in Table 4. For the air exchange rate for a shower stall, we used 0.2 min^-1^, also as shown in Table 2, which is near the value measured by Xu and Weisel [30] with the shower on, namely 0.14 ± 0.027 min^-1^.

### Humidifiers: Behavior models

Several parameters need to be quantified to describe behavior related to humidifier use: (1) the fraction of the population served by a WDS that uses a humidifier, (2) the type of humidifier being used, (3) the time when water is added to the humidifier reservoir, and (4) the length of time the humidifier is used. Values for all these parameters generally are not available. Consequently, sensitivity analysis is used to examine their importance.

Humidifier use is assumed to be distributed randomly in the population. Those individuals who use a humidifier are assumed to use it every day and one time per day. The only difference between the daily events for a particular simulated individual is that contaminant concentration in the water used to fill the humidifier reservoir generally will change during the course of a simulation. Humidifiers are assumed to be used in a bedroom while individuals are sleeping and the exposure period (time of use) is assumed to be 8 h. Units are assumed to be filled immediately before use and use begins at 22:00 hours.

Humidifiers are likely to be used primarily during periods of low humidity (use also is likely to vary regionally). Application of the approach presented here is most appropriate for such periods. However, season and location are not considered explicitly.

On the basis of surveys, about 15% of the households in the U.S. used a humidifier sometime during 2009, a small increase over the approximately 14% of households reporting the use of a humidifier in 1993 [57]. The surveys did not distinguish between the use of whole-house (furnace, central, forced-air) and portable humidifiers. However, about 96% of the humidifiers sold in the U.S. are portable units [57]. U.S. EPA reported that about 20% of portable units are ultrasonic and 50% are cool-mist (evaporative) humidifiers [57]. However, these estimates are not consistent with our very informal survey of portable units available for sale in the U.S., which suggests that a substantial portion of portable humidifiers being used are ultrasonic units. Manufacturers apply the label “cool mist” to ultrasonic, evaporative, and impeller humidifiers, which may lead to confusion when categorizing humidifiers. The U.S. EPA survey and our informal survey suggest that impeller humidifiers are not widely used. More definitive data on humidifier use are needed.

We assume conservatively that all 15% of the U.S. households that report using a humidifier use a portable unit and that a significant fraction of such households use an ultrasonic humidifier sometime during the year. Humidifier use likely is not uniform geographically, with higher use in some locations than in others. We use a base case in which 20% of potentially affected households use an ultrasonic humidifier and examine the sensitivity to this assumption.

### Humidifiers: Estimating inhalation doses

The approach utilized for estimating inhalation doses associated with the use of a humidifier is essentially the same as that employed for estimating inhalation doses associated with showering. However, the values of the parameters are different and transient effects are neglected. The discussion here considers ultrasonic humidifiers. The approach would be the same if impeller humidifiers were examined, except for differences related to the rate at which aerosols are generated and to the size distribution of the those aerosols. Both types of humidifiers readily aerosolize bacteria [37].

Ultrasonic humidifiers are very efficient generators of aerosol particles [33, 35, 36]. Therefore, even though the rate at which water is used in a humidifier is much less than that in a shower, the rate at which aerosols are generated can be larger. Because of the relatively small volume of water used in a humidifier, such use is less important as a source of volatile contaminants than showering. Showering can remove a sizable fraction of any volatile contaminants present and uses a much larger volume of water per event than does a humidifier.

Relative to showering, the duration of exposures to contaminants released by an ultrasonic humidifier can be very long. The average duration of a showering event is less than eight minutes. Exposures related to humidifier use can last many hours. Consequently, the transient effects associated with turning on and turning off a humidifier are less important than such effects are for showering. The approach presented here for humidifiers is based on the assumption that contaminant air concentration is constant and at its equilibrium value throughout the entire exposure period. Transient effects associated with turn-on and turn-off periods are neglected.

An ultrasonic humidifier is assumed to convert all water in the humidifier into inhalable aerosol particles. The only parameter needed to describe a humidifier is the rate at which water is used, which is assumed to be 0.5 L/h. Maintaining this rate for an 8-h period requires a humidifier with a capacity of 4 L or larger. In the U.S., many popular room humidifiers have such capacities and adequate output.

The parameters needed to describe the environment in which a humidifier is used are the volume of the room in which the humidifier and the receptor are located and the removal rate for the aerosols. We used values of 30 m^3^ (1,060 ft^3^) and 1.0 h^−1^, respectively, for these parameters. The volume is that of a moderately sized bedroom. The removal rate equals the air exchange rate, which, for a bedroom, is assumed to be twice that for the residence, plus the settling rate for the aerosols. U.S. EPA’s recommended air exchange rate for residences for exposure assessments is 0.45 h^−1^, which is the median value across all U.S. census regions [58]. Few measurements have been reported for air exchange rates for bedrooms [59]. A study that reported measured air exchange rates for bedrooms found a median value of 1.23 h^−1^ (N = 253), with a median volume of 25 m^3^ for the rooms [59]. The study also reported a median value for air exchange rates for the residences of 0.57 h^−1^ and noted that, overall, air exchange rates for the bedrooms were about twice those for the residences studied [59]. A settling rate of 0.14 h^−1^ has been estimated for fine particles from ultrasonic humidifiers [33]. Combining twice the recommended air exchange rate for residences with the estimated settling rate for fine particles gives a rate of about 1.0 h^−1^, which we use as an approximation of the aerosol removal rate for a bedroom.

A mass-balance model was used to determine the air concentration of the contaminant. It assumes complete mixing of the contaminant in the air of the room and neglects transient effects. The inhalation dose, *D*_*h*_ (mass or number of cells) for one humidifier event is

$$\begin{array}{c} D_{h}=\frac{BC_{h}G_{h}T_{h}}{kV_{h}} \end{array}$$ (7)

where *C*_*h*_ is the contaminant concentration in the humidifier water (mg/L or cells/L), *G*_*h*_ is the aerosol volume generation rate (0.5 L/h), *T*_*h*_ is the duration of exposure (8 h), *k* is the removal rate for aerosols (1.0 h^−1^), and *V*_*h*_ is the volume of the room (30 m^3^).

As noted above, the aerosol volume generation rate is assumed to be equal to the water use rate; all water is assumed to be converted into inhalable aerosols and released into the room. The contaminant concentration in the humidifier water equals the contaminant concentration in the water in the distribution system at the location of the receptor at the time the water is withdrawn for use in the humidifier.

The breathing rate (*B*) needs to be consistent with the assumed use of the humidifier. If the humidifier is used while sleeping, then the appropriate breathing rate is the average short-term rate while sleeping, which is about 0.005 m^3^/min, for both children and adults [56].

### System-wide consequences

The cumulative number of simulated individuals receiving inhalation and ingestion doses above various dose levels were estimated for each scenario and statistics for impacts were then determined considering all scenarios in an ensemble. For example, after estimating impacts associated with all injection nodes, the 95th percentile impact for inhalation for a network was determined for a dose level of 1 mg. The conditions in the network are the same for all scenarios. Only the injection location changes. In general, the injection location associated with a particular percentile impact is not the same for different dose levels. For example, the injection node that is associated with the 95th percentile impact at a dose level of 1 mg is generally not the same as the node associated with the 95th percentile impact at a dose level of 0.1 mg.

## Application and Discussion

We determined impacts associated with the inhalation of contaminants during showering and humidifier use for three water distribution systems. For comparison, we also determined impacts associated with the ingestion of tap water. Descriptions of the network models for the three systems are given in Table 5. All three can generally be classified as looped networks. Networks E1 and E3 are primarily groundwater-supplied systems, while Network E2 uses surface water. Networks E1 and E3 represent relatively flat topographical areas. Networks E2 serves a community challenged by significant topographical diversity. The network models for the three systems are provided in S1 Data.

**Table 5:** Network Descriptions.

Quantity	Network		
	E1	E2	E3
Population (10^3^)	75	150	250
Mean water use (m^3^/s)	0.4	1.3	1.4
Per capita use (L/d)	430	760	480
Area (km^2^)	78	500	490
Nodes (10^3^)	3.2	3.4	13
NZD nodes (10^3^)	3.0	1.6	11
Pipes (10^3^)	3.9	3.8	15
Tanks	7	34	2
Reservoirs	10	1	2
Pumps	24	61	4
Valves	17	2	5
Mean population, NZD nodes	25	94	24
Median population, NZD nodes	17	76	16

All numbers are rounded independently to two significant figures. NZD, non-zero demand.

All of these networks have been used in previous studies. We have used Networks E1, E2, and E3, labeled as Networks 2, 4, and 6, respectively [8], and Network E3, labeled as Network N1 [60]. Networks E2 and E3 were used by Watson et al. [61] as their Network2 and Network3, respectively; they have made the models for these two networks available. Network E3 was used by Klise et al. [62], who labeled it BWSN and by Ostfeld et al. [63], who called it Network 2.

**Fig 6:**
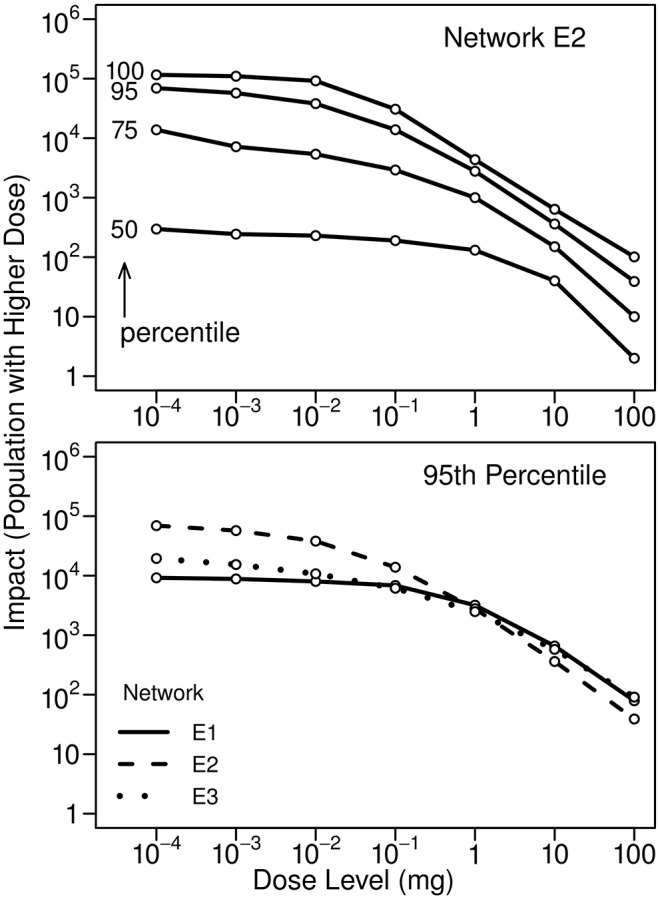
Impacts associated with inhalation of a volatile contaminant during showering. The upper figure shows 50th to 100th percentile impacts for Network E2. The lower figure compares 95th percentile impacts for the three networks. Ten kilograms of contaminant was injected at 0:00 hours for all three networks.

The upper plot in Fig 6 illustrates, for Network E2, how impacts associated with the inhalation of a volatile contaminant during showering vary with dose level for different impact percentiles. (Note that the 100th percentile, or maximum, impact shown in the figure is not a worst-case impact. The times when individuals shower and the durations of their showers are assigned randomly. Estimates of worst-case conditions would require non-random assignment of these quantities.) The lower plot in the figure compares 95th percentile impacts for the three networks. Impacts plateau at low dose levels because they are limited by the size of the population that can be exposed; even at extremely low concentrations, the contaminant does not reach a significantly larger portion of the networks. At high dose levels impacts tend to be similar for all networks because only a relatively small portion of a network experiences the high contaminant concentrations needed to obtain such high doses. At such small spatial scales, all of the networks tend to be similar. Note that the plots in the figure use logarithmic scales on both axes.

**Fig 7:**
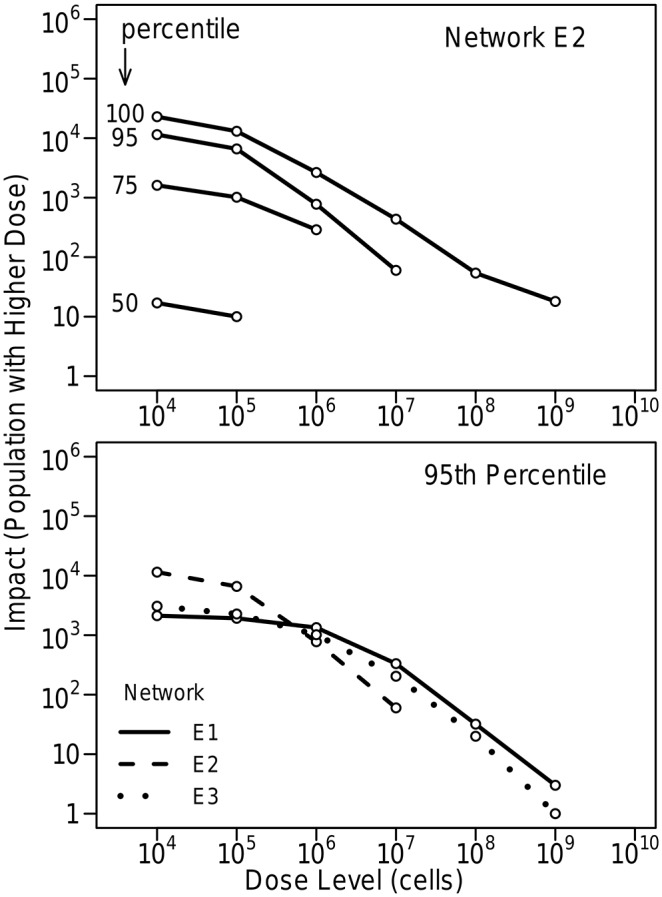
Impacts associated with inhalation of a microbial contaminant due to ultrasonic humidifier use. The upper figure shows 50th to 100th percentile impacts for Network E2. The lower figure compares 95th percentile impacts for the three networks. Ten kilograms of contaminant, with 10^8^ cells/mg, were injected at 0:00 hours for all threes networks. Twenty percent of the population is assumed to use humidifiers. Humidifiers are filled at 22:00 and used for 8 hours.

The upper plot in Fig 7 illustrates, again for Network E2, how impacts associated with inhalation of a microbial contaminant as the result of (ultrasonic) humidifier use vary with dose level for different impact percentiles. The lower plot compares 95th percentile impacts for the three networks. Impacts behave similarly to those shown in Fig 6, but are lower because a much smaller portion of the population uses humidifiers than showers.

To help put these results in perspective, Table 6 provides estimates of the contaminant quantities generated by showering and humidifier use and the potential inhalation doses received by an individual engaged in those activities using contaminated water. These estimates are not based on simulations of contaminant transport; they use the approaches for estimating doses developed above and simply assume that contaminant concentrations in the water being used are either 1 mg/L for a chemical contaminant or 10^8^ cells/L for a microbial contaminant. Relative to the inhalation dose received due to humidifier use or due to inhalation of volatile contaminants while showering, the dose due to inhalation of chemically contaminated aerosols while showering is small. This small dose is a consequence of the relatively inefficient generation of aerosols in a shower. An ultrasonic humidifier converts nearly 100% of the mass of water in the humidifier to aerosols. Depending on the contaminant, a shower can strip as much as 80% of a volatile contaminant from the shower water. However, a shower with a flow rate of 9 L/min generates aerosols at a rate of only about 6 mg/min or less (see Table 3), an aerosol generation efficiency of about 100 ×(6 mg/9 kg) or 6.7 × 10^-5^% or less. For comparison with the inhalation doses for nonvolatile contaminants given in Table 6, ingestion of 1.0 L of water with a contaminant concentration of 1 mg/L or 10^8^ cells/L results in an ingestion dose of 1.0 mg or 10^8^ cells. For nonvolatile chemicals, ingestion is likely to be a more important pathway for exposure than inhalation, in terms of the size of the dose.

**Table 6:** Illustrative Potential Inhalation Doses from Showering and Humidifier Use.

Activity	Aerosol Mass (mg)	Contaminant Released		Inhalation Dose	
		(mg)^a^	(cells)^b^	(mg)^a^	(cells)^b^
Showering (vapors)^c^	-	58	-	1.2	-
Showering (aerosols)^c^	48	4.8 × 10^-5^	4.8 × 10^3^	1 × 10^-6^	1 × 10^2^
Humidifier use (aerosols)^d^	4 × 10^6^	4	4 × 10^8^	0.04	4 × 10^6^

^a^ Contaminant concentration in water is 1 mg/L.

^b^ Contaminant concentration in water is 10^8^ cells/L.

^c^ Parameters from Table 2. Eight-minute shower, 2-minute exposure after shower is turned off.

^d^ Parameters from Sec. 2.6. Exposure period is 8 h; 0.5 L/h water use.

All impacts presented here can be scaled to accommodate injection masses different from the ones used in this study. For example, the plots in Fig 6 were obtained using an injection mass of 10 kg. For different injection masses, the curves in the plots have the same shapes; however, the scale on the horizontal axis must be modified. For an injection mass of 1 kg, the values on the horizontal axis need to be multiplied by 0.1. For an injection mass of 100 kg, they need to be multiplied by 10. If desired, similar adjustments can be made for the other impacts presented here.

### Showering

**Fig 8:**
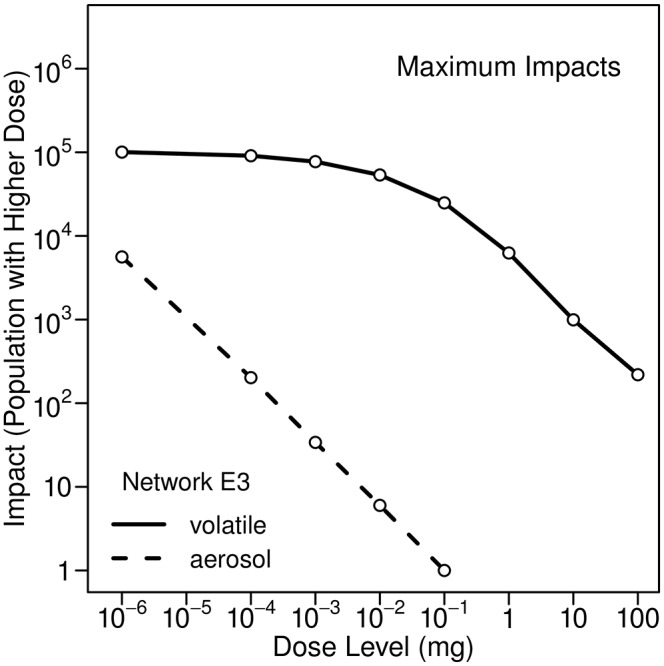
Maximum impacts from inhalation of volatile and nonvolatile contaminants during showering. Results are for Network E3 from two separate simulations, both using 10-kg injections at 0:00 hours. Nonvolatile contaminants are in aerosols in the shower air.

During showering, individuals can inhale volatile or nonvolatile contaminants, or both. Nonvolatile contaminants in shower air are associated with aerosols and can be chemical or microbial in nature. Given the same contaminant concentration in water, inhalation doses of volatile contaminants will be much larger than inhalation doses of nonvolatile contaminants, as discussed above. A comparison of maximum impacts (100th percentile) for Network E3 for volatile and nonvolatile contaminants is provided in Fig 8. Although the inhalation dose of a nonvolatile contaminant is much less than the inhalation dose of a volatile contaminant if both have the same concentration in water, it is possible for a relatively small number of individuals to receive a significant inhalation dose of a microbial contaminant. For example, from the figure, about 200 individuals receive a dose of 10^-4^ mg or larger due to inhalation of aerosols. For a microbial contaminant this could be equivalent to 10^4^ cells or more, using our assumption concerning the concentration of microbial contaminants in the injected mass. Showering could result in significant localized consequences due to exposures to microorganisms with a low infectious dose. Considering only a single injection node and using only a short 24-h simulation, Schijven et al. [9] reached a similar conclusion, namely that showering could be a relevant source of exposure to highly infectious microorganisms, as noted above.

The results presented here are based on the assumption that the time simulated individuals remain in the shower stall after the shower is turned off is two minutes (*t*_2_ − *t*_1_ = 2). As noted above, there appears to be no information available on the time individuals actually do spend in the shower stall after turning off the shower. If *t*_2_ − *t*_1_ = 0, 1, or 3 minutes instead of 2 minutes, the average individual inhalation dose of a volatile contaminant for a population whose shower durations are distributed lognormally, as discussed above, changes by about -23, -10, or +8%, respectively, assuming the contaminant concentration is the same for all individuals. For the same changes in *t*_2_ − *t*_1_, the 95th percentile individual inhalation dose of a volatile contaminant changes by about -13, -6, and +5%, respectively. Given the logarithmic scales used in plots of impacts versus dose, such as those in Fig 6, little change would be noticed if *t*_2_ − *t*_1_ = 0, 1, or 3 minutes were used instead of 2 minutes.

### Humidifier use

For volatile chemicals, the inhalation dose that an individual might receive from ultrasonic humidifier use is likely to be smaller than that associated with showering, as shown in Table 6. However, for microbial contaminants, inhalation doses can be much larger. The focus here is on microbial contaminants.

**Fig 9:**
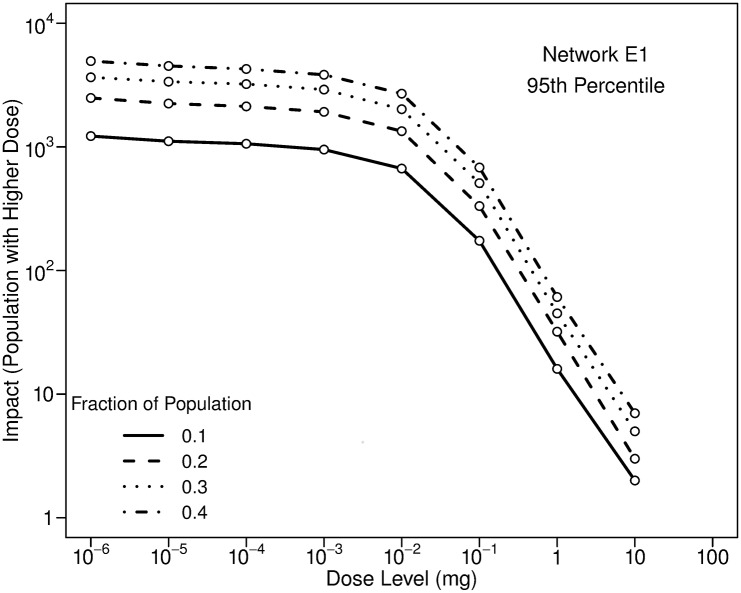
Influence of the fraction of the population using ultrasonic humidifiers on 95th percentile impacts. Results are for Network E1, an injection at 0:00 with a mass equal to 10 kg, and a fill time for all humidifiers of 22:00. Humidifiers are used for eight hours each day. The different lines in the plot provide results for different fractions of the population that use humidifiers.

A substantially smaller fraction of the population uses portable humidifiers than showers. Fig 9 illustrates the sensitivity of impacts to the portion of the population that uses ultrasonic humidifiers. As would be expected, impacts increase with the fraction of the population using humidifiers. Uncertainties in estimates of impacts can be large, given considerable uncertainty in the fraction of the population that uses humidifiers for a particular WDS.

**Fig 10:**
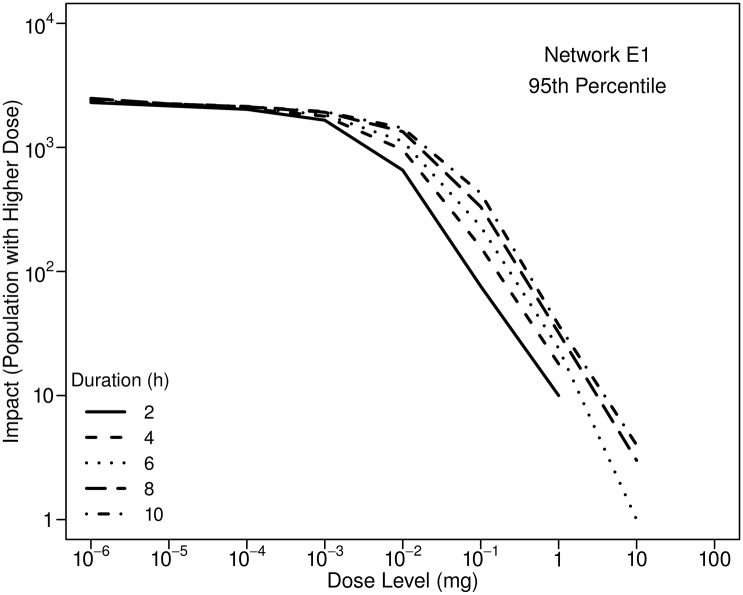
Influence of the daily duration of humidifier use on 95th percentile impacts. Results are for Network E1, an injection at 0:00 with a mass equal to 10 kg, and a fill time for all humidifiers of 22:00. Ultrasonic humidifiers are assumed to be used by 20% of the population.

Dose increases with exposure duration. As is the case with the fraction of the population using humidifiers, duration of use is not well quantified. Fig 10 shows how estimated impacts are affected by duration of humidifier use. At higher doses, impacts are sensitive to how long the humidifiers are used. (Note in the figure that the curve for a duration of 2 h, for example, does not extend to values of dose greater than 1 mg. This apparent abrupt termination of curves in figures such as Fig 10 is a consequence of the logarithmic scale used on the vertical axis. For a duration of 2 h, the impact for a dose level of 10 mg is zero and, therefore cannot be shown on the plot.)

**Fig 11:**
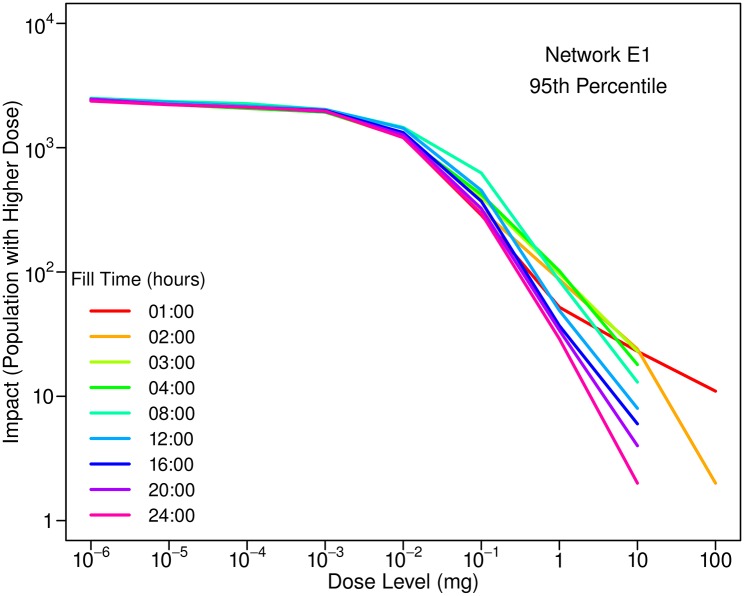
Influence of the time when humidifiers are filled on 95th percentile impacts. Results are for Network E1 and an injection at 0:00 hours with a mass equal to 10 kg. Ultrasonic humidifiers are assumed to be used for eight hours each day by 20% of the population. Note that this figure should be viewed in color.

The times at which humidifiers are filled with water do not appear to have been studied. Fig 11 illustrates the sensitivity of impacts to the time when humidifiers are assumed to be filled. At higher doses, impacts are sensitive to fill time. When humidifiers are filled shortly after a contaminant is injected (see fill times of 01:00 and 02:00 hours in the figure), some individuals located near the injection location could receive a relatively large dose of contaminant.

**Fig 12:**
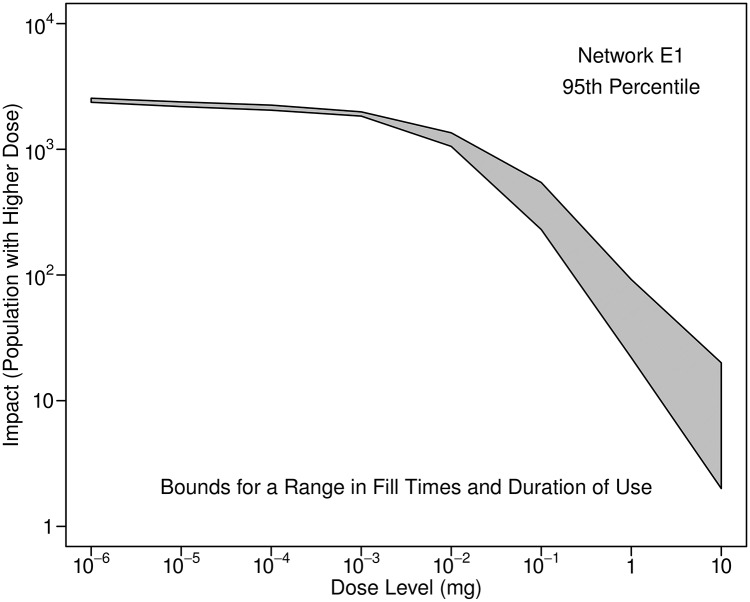
Bounds on estimated 95th percentile impacts associated with humidifier use. The humidifier can be filled at any time and the daily duration of use can have any value from 2 to 10 hours. Results are for Network E1 and an injection at 0:00 with a mass equal to 10 kg. Twenty percent of the population is assumed to use ultrasonic humidifiers.

Impacts associated with humidifier use were estimated assuming that fill times for humidifiers are equally likely to occur anytime throughout the day and that the duration of use is uniformly distributed from 2 to 10 hours. The range in estimated 95th percentile impacts that results is illustrated in Fig 12, which shows the bounds on impacts for this range in fill time and duration of use.

**Fig 13:**
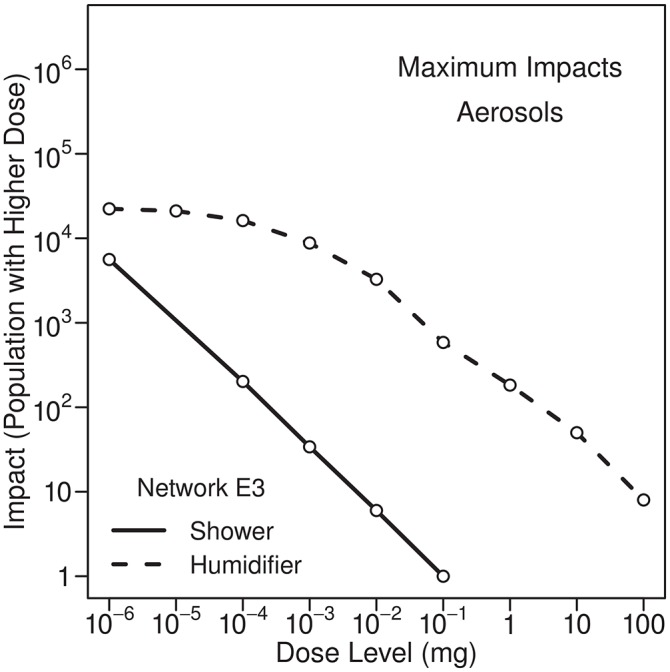
Maximum impacts associated with inhalation of contaminated aerosols during showering and humidifier use. Results are for Network E3 and an injection at 0:00 with a mass equal to 10 kg. Ultrasonic humidifiers are assumed to be filled at 22:00 hours and used for eight hours each day by 20% of the population.

As discussed above, the dose associated with the inhalation of contaminated aerosols from humidifier use is generally expected to be much larger than that from showering. Fig 13 compares the maximum impacts associated with the inhalation of contaminated aerosols from showering and humidifier use for Network E3; the impact from humidifier use is much larger at all dose levels.

### Variability in results due to probabilistic behavior models

The use of probabilistic models for behavior produces variability in estimated impacts. Table 7 summarizes the variability in estimated impacts resulting from the use of these models. The table gives relative standard deviations in estimated 95th percentile impacts for different dose levels. The relative standard deviations were determined using a sample of 100 independent ensembles of injection scenarios for Networks E1 or E2. The results in the table show that the variability in impacts from ensemble to ensemble is small. This is expected because an impact is the number of persons receiving a dose above some level obtained by summing across all downstream nodes for an injection location, a process that tends to result in the cancellation of independent variations at individual nodes. Given the small variability in impacts for different ensembles, the results presented in this paper were obtained using a single ensemble of impacts. Results would change little if averages obtained from many ensembles were used instead, as demonstrated by the small relative standard deviations in Table 7. However, the use of only a single ensemble yields a considerable saving in computational time.

**Table 7:** Variability in 95th Percentile Impacts Due to the Probabilistic Nature of Behavior Models.

Dose Level (mg)^b^	Relative Standard Deviation (%)^a^			
	Inhalation			Ingestion
	Showering		Humidifer Aerosols^c^	
	Volatiles	Aerosols		
10^-4^	0.2	3.0	0.8	<0.1
10^-3^	0.2	^d^	1.2	0.1
10^-2^	0.3	^e^	1.0	0.1
10^-1^	0.5	^e^	1.7	0.4
1	0.7	^e^	2.8	0.6
10	1.5	^e^	^f^	1.3
10^2^	2.5	^e^	^e^	3.7

^a^Results are for Network E2, except for humidifiers, for which results are for Network E1. The relative standard deviations in impacts were determined from 100 independently simulated ensembles of injections of a 10-kg mass of contaminant at 0:00 hours.

^b^For aerosols and ingestion, multiply by 10^8^ cells/mg to get a value for dose in number of cells.

^c^Ultrasonic humidifiers are assumed to be used for eight hours each day by 20% of the population.

^d^For the 100 ensembles, the range in values of the 95th percentile impact is from 1 to 2. The absolute variability is very small.

^e^Impact is zero.

^f^For the 100 ensembles, the range in values of the 95th percentile impact is from 3 to 4. The absolute variability is very small.

### Contaminant mass balances

Conservation of mass requires that the mass of a contaminant removed from a WDS during a simulation (*R*) plus the mass of the contaminant still in tanks and pipes in the system at the end of the simulation (*T* + *P*) equal the mass of the contaminant injected into the system (*I*). However, mass is not always conserved during water-quality simulations with EPANET [60]. Such mass imbalances generally appear to be the result of using a water-quality time step in the simulation that is too long for the network being considered. The default water-quality time step for EPANET is 300 s. We used a time step of 60 s. However, for some networks noticeable mass imbalances occur with a time step as short as 1 s, which is the minimum water-quality time step that can be used in EPANET. Use of such a short time step can result in unacceptably long times to complete a simulation. Although reducing the water-quality time step generally improves mass balances, it does not always have a significant effect on 95th and 100th percentile impacts, which are used in this study to quantify consequences of contamination events.

For the three networks considered, Table 8 shows the effect on contaminant mass balance of reducing the water-quality time step used in EPANET simulations from 60 s to 1 s. For each network, results are summarized for an ensemble of injection scenarios; mass balances were determined separately for each scenario. Mass imbalances are substantially reduced with the shorter time step; they are essentially eliminated for Networks E1 and E3. Obtaining acceptable mass balances for Network E2 would require a time step well below 1 s. Table 9 shows how impacts at the 95th and 100th percentile levels are affected when the water-quality time step is reduced from 60 s to 1 s. Impacts were determined using a non-probabilistic model for ingestion [6] that assumes the same quantity of water is ingested at five fixed times during the day. Use of this model facilitates comparisons: it eliminates the randomness associated with the inhalation models presented here and distributes the exposures throughout the day. Ninety-fifth and 100th percentile impacts obtained with this model were compared at seven dose levels (10^-4^, 10^-3^, …, 10, 100 mg). Reducing the time step has only a small effect on these impacts, with the average difference in impacts generally less than 1%. Therefore, although using a 1-s time step with the three networks provides considerable improvement in mass balance, it has relatively little benefit for estimates of the these impacts. Consequently, we expect that use of a 60-s water-quality time step in our simulations provides acceptable estimates of inhalation impacts, although some sizable mass imbalances may occur.

**Table 8:** Contaminant Mass Balances Obtained with 60- and 1-s Water-quality Time Steps^a^.

Quantity	Network E1		Network E2		Network E3	
	60 s	1 s	60 s	1 s	60 s	1 s
Minimum mass-balance ratio^b^^c^	0.978	0.993	0.828	0.895	0.980	1.000
Maximum mass-balance ratio	1.131	1.005	1.060	1.006	1.018	1.000
Scenarios (%) with imbalance > 0.1%^d^	22.9	0.2	42.7	20.6	9.4	0.0
Scenarios (%) with imbalance > 1.0%	3.1	0.0	18.2	10.5	0.3	0.0
Scenarios (%) with imbalance > 10.0%	<0.1	0.0	0.1	<0.1	0.0	0.0

^a^Results are for an ensemble, with a 168-h simulation and contaminant injected during the first hour.

^b^Mass-balance ratio = (*R*+*T*+*P*)/*I*. The minimum ratio is the value for the scenario with the smallest mass-balance ratio.

^c^Three deadend nodes in Network E3 have no flow at the time contaminant is injected. As a result, the contaminant is lost and the mass-balance ratio is zero. This value is not used as the minimum mass-balance ratio for the network.

^d^Mass imbalance = |(mass-balance ratio—1)|.

**Table 9:** Differences in 95th and 100th Percentile Impacts Obtained with 60- and 1-s Water-quality Time Steps.^a^.

Quantity	Network E1		Network E2		Network E3	
	95th	100th	95th	100th	95th	100th
Maximum relative difference (%)^b^	0.3	2.5	6.4	1.0	0.9	3.7
Average relative difference (%)	0.1	0.8	1.9	0.2	0.4	0.9

^a^For seven dose levels (from 10^-4^ to 100 mg, by decade). Impacts were obtained using a non-probabilistic model for ingestion that assumes individuals drink the same amount of tap water at five fixed times during the day. Results are for an ensemble, with a 168-h simulation and contaminant injected during the first hour.

^b^Relative difference in impacts equals the absolute value of the difference between the impacts obtained for a particular dose level using 1-s and 60-s water-quality time steps divided by the impact obtained using a 60-s time step.

Simulations were performed on a server with four 2.3-GHz processors, utilizing a total of 32 cores, a configuration that can perform 32 simultaneous EPANET and exposure simulations. For a 1-s EPANET water-quality time step, the simulation of one scenario requires about 20 min for Networks E1 and E2 and 70 min for Network E3. Therefore, the time required to complete an ensemble of non-zero demand nodes is about 31 h for Network E1, 17 h for Network E2, and 16 days for Network E3. Given the long execution times required for simulations using a 1-s water-quality time step and the small difference expected between impacts determined using 1-s and 60-s time steps, we used a 60-s water-quality time step in our simulations.

### Establishing bounds on impacts

Impacts related to inhalation of contaminants are constrained by the mass of contaminant injected into the WDS and the population served by the system. Impacts cannot exceed the size of the potentially exposed population. At large doses, the size of impacts is limited by the mass of contaminant in the system.

Detailed descriptions of the three WDSs considered in this study were needed to perform simulations. Such detailed information is not available for all distribution systems. However, in such cases, there may still be an interest in assessing the consequences of a contamination event. Bounds on impacts can be obtained using relatively little information and may be of value when such an assessment is needed. Simple bounds on impacts associated with inhalation of contaminants during a contamination event are developed in the Appendix. Such bounds have previously been developed for impacts associated with ingestion of tap water [8].

The upper bound on impacts associated with showering is established by the size of the potentially exposed population. For showering, about 22% of the population is not exposed because they do not shower. Therefore, the upper bound on impacts is 0.78 times the size of the population.

**Fig 14:**
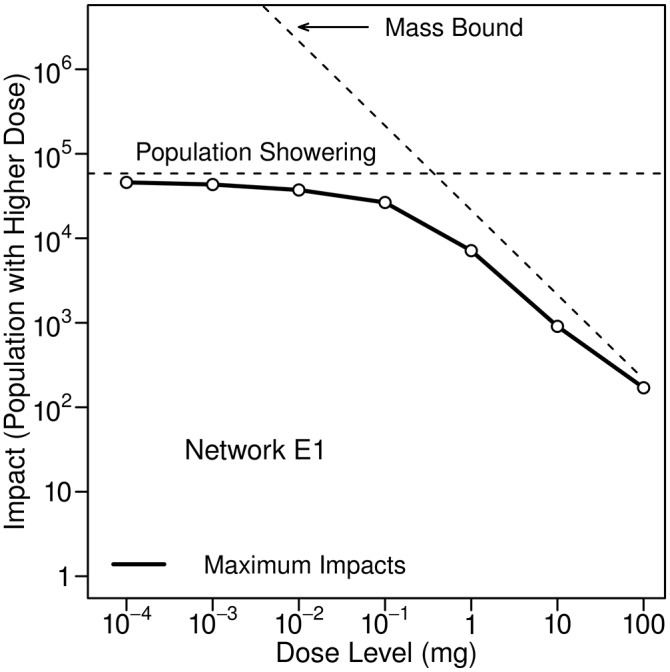
Example of bounds on inhalation impacts associated with showering. Results are for Network E1 and 10 kg of a volatile contaminant injected at 0:00 hours.

Fig 14 illustrates the application of bounds on inhalation impacts associated with showering for a volatile contaminant using results for Network E1. At low doses, maximum (100th percentile) impacts approach the size of the potentially exposed population. At high doses, maximum impacts approach the mass-based bounds, which are given by Eq (A7) in the Appendix.

The development of mass-based bounds on inhalation impacts presented in the Appendix uses an average shower duration and implicitly assumes that the ratio of water used for showering and for all purposes is a constant. Shower duration actually varies from simulated individual to individual and the ratio of water used for showering and for all purposes can vary both spatially and temporally. Therefore, in some cases refinement of the simple mass-based bounds developed here may be necessary.

**Fig 15:**
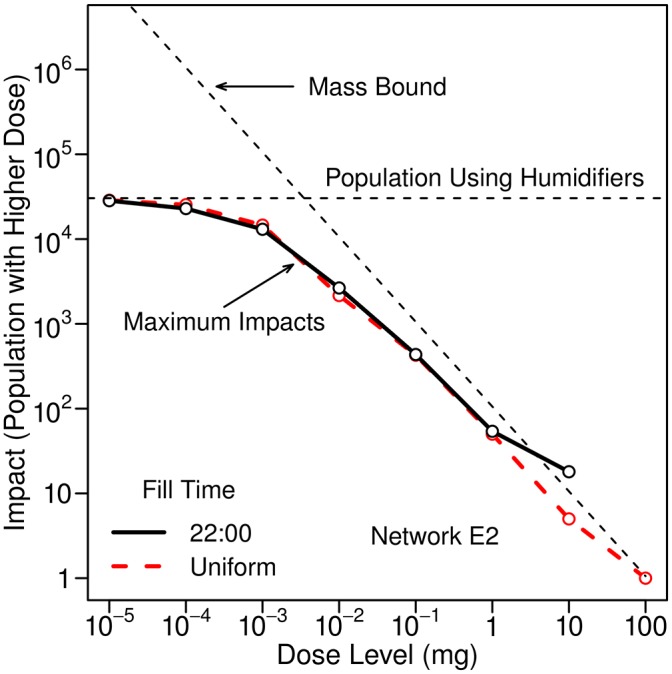
Example of bounds on inhalation impacts associated with humidifier use. Results are for aerosols for Network E2 and 10 kg of contaminant injected at 0:00 hours. Twenty per cent of the population is assumed to use ultrasonic humidifiers for eight hours each day, with fill times either at 22:00 hours or uniformly distributed from 06:00 to 23:30 hours.

As with showering, the upper bound on impacts associated with humidifier use is established by the size of the exposed population. We assume that 20% of the population uses ultrasonic humidifiers, which establishes the upper bound on impacts. Fig 15 shows the application of bounds on inhalation impacts associated with humidifier use for Network E1. The mass-based bound for impacts for humidifier used are given by Eq (A13) in the Appendix. The bound shown in Fig 15 assumes an injection mass of 10 kg, humidifier water use of 4 L/d, and per capita water use for all purposes of 757 L/d (200 gal/d).

The plot in Fig 15 shows that the maximum (100th percentile) impact exceeds the mass-based bounds at a dose level of 10 mg when it is assumed that all humidifiers are filled at 22:00 hours. The mass-based bounds were developed using a number of assumptions. If any of these assumptions is not satisfied, the bound can be exceeded. In such cases, bounds based on more realistic assumptions are needed. There are at least two assumptions that may not be satisfied. First, as noted above, the bounds on impacts associated with humidifier use implicitly assume that the ratio of the rate at which water is used in humidifiers to the total rate at which water is used (*q*_*h*_/*Q*) is constant and does not vary over the period of interest. This assumption may not always be valid and in such cases impacts could exceed the mass-based bound given by Eq (A13). It is unlikely that the assumption of a constant ratio is valid when it is assumed that all humidifiers are filled at 22:00 hours. The ratio is zero, except at this time, at which it will exceed the average value of the ratio for the day. Second, each simulated individual using water from the network is assumed to use a humidifier with a probability of 0.2. Across a large population, 20% of simulated individuals will use ultrasonic humidifiers. However, in a small area of the network the portion of the population using humidifiers can be quite different. The portion of the network over which individuals can potentially inhale large doses of contaminants (e.g., 10 mg) as a result of humidifier use is small and consequently, for a particular scenario, the fraction of these individuals who use humidifiers can differ considerably from the network average. For Network E2, the simple bound at 10 mg is exceeded because for the 100th percentile injection node the contaminant concentration at an adjacent node is elevated at 22:00 hours when simulated individuals at that node fill humidifiers. The assumption of a constant ratio for *q*_*h*_/*Q* is not satisfied. However, when it is assumed that the fill time for humidifiers is uniformly distributed from 06:00 to 23:30 hours, a situation for which the assumption of a constant ratio is more likely to be satisfied, the maximum impacts do not exceed the mass bounds, as is shown in Fig 15.

If needed for a specific application, improved mass-based bounds can be developed that rely on fewer assumptions. An example of the development of such bounds is available for ingestion [8].

### Consideration of whole-house humidifiers

Whole-house (furnace) humidifiers can potentially release contaminants to the air in a dwelling. Given the volume of water used and the nature of such humidifiers, they are generally expected to be less important as a source of contaminants than showering or portable humidifier use. There are various types of whole-house humidifiers. Common types used in the U.S. add moisture to the air by evaporating water or by generating steam. Given their design it seems unlikely that they are efficient sources of aerosols. However, we are not aware of any studies that have examined aerosol generation by whole-house humidifiers. Here they are examined as possible sources of both volatile and aerosol-borne contaminants.

Consider a simple model in which one compartment is used to represent a dwelling and a contaminant is introduced into the compartment via a forced-air system that results in the contaminant being well mixed throughout the compartment. An inhalation dose can be estimated using either Eq (4) or Eq (6), assuming that the exposure period is long and that transient effects associated with starting and stopping the humidifier can be neglected. In this case, the inhalation dose *D* is given by

$$\begin{array}{c} D=\frac{BGt_{d}}{\lambda V_{d}} \end{array}$$ (8)

where *B* is the breathing rate, *G* is the generation rate for a contaminant for the humidifier, *t*_*d*_ is the exposure period, *λ* is the removal rate for the contaminant, and *V*_*d*_ is the volume of the dwelling.

Eq (8) is used in the following paragraphs to estimate inhalation doses for contaminants for a particular humidifier model that is popular in the U.S. This model evaporates about 2.7 L of water per hour and has a feed rate of about 11 L/h. The humidifier distributes about 0.2 L/min of water uniformly across the top end (about 25-cm wide) of a vertical evaporative pad, about 4-cm thick and 30-cm long. Hot air flows through the pad and evaporates water. The water vapor is then transported through the ventilation system to the interior of the dwelling.

#### Volatilization

For volatile contaminants, the generation rate equals the rate at which water is evaporated multiplied by the contaminant concentration in the water. Using the evaporation rate for the humidifier mentioned above, 2.7 L/h, and a contaminant concentration of 1 mg/L, the generation rate is 2.7 mg/h. For a 24-h period, the contaminant mass released (*Gt*_*d*_) is 65 mg. (From Table 6 the mass of a volatile contaminant released during a typical shower using water with the same contaminant concentration is 58 mg.) All of the contaminant in the water being evaporated is assumed to be distributed by a forced-air system to the single compartment of a dwelling.

For volatile contaminants, the removal rate equals the air exchange rate. Recommended values for dwelling volume and air exchange rate for use in risk assessments are available [58]. For conservative assessments, 10th percentile values for dwelling volume and air exchange rate are 154 m^3^ and 0.18 air exchanges per hour, respectively. For a more typical case, the mean volume is 492 m^3^ and the median air exchange rate is 0.45 h^-1^.

Assuming that the contaminant concentration in the water used is constant for 24 h, that the humidifier operates without stopping for 24 h, and that an individual remains in the dwelling for the entire period, the dose (*D*) is 140*B* mg for the conservative case and 17.5*B* mg for the more typical case, where *B* has units of m^3^/min. The short-term average breathing rate for light activity is about 0.012 m^3^/min and for sedentary activity is about 0.005 m^3^/min [56]. Therefore, for the conservative case the estimated daily inhalation dose of a volatile contaminant could vary from about 0.7 to about 1.7 mg, depending on the level of activity, and for a more typical case could vary from about 0.09 to about 0.2 mg. Note that both of these cases are rather conservative, requiring that the contaminant be present in the water feeding the humidifier for 24 h, that the humidifier run without interruption for 24 h, and that the individual receiving the dose be present for 24 h (and for the higher doses for each case also be active). Comparing these estimates with the estimate in Table 6 for the potential inhalation dose for volatiles for a single showering event (1.2 mg) shows that, using a simple model for exposure for the humidifier and a very conservative set of assumptions, the estimated potential inhalation doses from showering and whole-house humidifier use could be similar. For more typical conditions for the whole-house humidifier, the estimated dose would be about an order of magnitude less than that estimated for showering, although the estimate is still based on the conservative assumptions that the contaminant is present continuously in the water and the humidifier never stops. If a more conservative approach is used to estimate the potential inhalation dose from showering, for example by assuming two long showers per day in a more poorly ventilated stall, the estimated dose could be increased by an order of magnitude.

For a risk assessment focused on reasonable worst-case conditions, whole-house humidifiers may be worth consideration as a source of volatile contaminants. However, for an assessment that is focused on the entire population, such as the current study, the potential effects associated with volatilization of contaminants by whole-house humidifiers appear to be small relative to those associated with showering.

#### Aerosolization

For aerosols the generation rate for a contaminant equals the rate at which aerosols are generated multiplied by the concentration of the contaminant in the aerosols. The removal rate is the sum of the air exchange rate and the deposition rate. As noted above, we have no estimates for the aerosol generation rate for whole-house humidifiers. However, the generation rate is expected to be much less than for showers. The flow rate for the whole-house humidifier being considered is about 0.2 L/min (11 L/h), while a typical shower might have a flow rate of 9 L/min through a nozzle that generates a spray. The flow rate for the shower is about 50 times that for the humidifier and the humidifier is less likely to generate aerosols than the shower. However, to obtain a conservative estimate for inhalation dose for the humidifier, assume that the humidifier is as efficient as a shower in generating aerosols and that the removal rate for aerosols equals the air exchange rate, neglecting any deposition in the ventilation ducts, filter, or dwelling. As discussed above, a shower can generate about 6 mg of aerosols per minute. For a flow rate of 9 L/min, the relative rate of aerosol generation is about 6.7 × 10^−7^ kg/kg. Assuming a bacterial concentration in the aerosols of 10^8^ cells/L, a 24-h exposure period, and the same dwelling volumes and air exchange rates as used for volatile contaminants, the inhalation dose for aerosols contaminated with bacteria is 38000*B* cells for a conservative dwelling volume and air exchange rate and 4800*B* cells for a more typical volume and air exchange rate.

Using the same breathing rates as for volatile contaminants, the inhalation dose ranges from about 190 to about 460 cells for the conservative case and from about 24 to about 58 cells for the more typical case. For comparison, from Table 6, using the same concentration of bacteria the inhalation dose for a single, typical shower is about 100 cells. For typical conditions, the calculated dose for the humidifier is less than that calculated for the shower, which is much less than that estimated for an ultrasonic humidifier. Considering the very conservative assumptions used to obtain the estimate for the whole-house humidifier (aerosol generation efficiency equal to that for a shower, deposition of aerosols neglected, contaminant present for 24 h, humidifier operating for 24 h without stopping), potential doses of aerosol-borne contaminants released by whole-house humidifiers are likely to be negligible relative to those associated with the use of ultrasonic humidifiers, particularly when considering population-wide effects.

### Interpretation of dose estimates

The doses calculated in this paper are potential, or intake, doses, i.e., the quantities of a contaminant inhaled as the result of showering or humidifier use. For chemical contaminants, the applied dose, the amount of contaminant that is present at a primary absorption barrier, such as the lining of the lung, is commonly assumed to equal the potential dose for contaminants entering the body by the respiratory route [64]. This assumption is generally necessary because of a lack of the information needed to properly determine the applied dose. The absorbed, or internal, dose, which is the quantity of contaminant that crosses the absorption barrier, equals the applied dose multiplied by an absorption fraction (AF) [64]. When AF equals 1, absorbed dose is approximately equal to the potential dose.

AF varies for different volatile chemicals. For example, Xu and Weisel [65] studied inhalation exposures to disinfection by-products during showering and found that about 85-90% of the haloketones inhaled during showering were absorbed, while only about 70% of the inhaled chloroform was absorbed. Determination of absorbed dose using chemical-specific values for AF requires separate calculations for each specific contaminant. Use of an AF equal to 1 for all contaminants considered in a study simplifies the analysis and provides conservative estimates of absorbed dose. For example, Kim et al. [66] considered various chemical contaminants and sources of exposure to these contaminants in drinking water, including showering, and assumed a value for AF of 1 for all chemicals. Given that we do not address specific contaminants, an AF equal to 1 can be assumed for volatile chemicals and our results interpreted conservatively in terms of absorbed doses.

For contaminants present in aerosols, determination of applied dose is complicated by the influence of particle size on the deposition of the contaminated aerosols. Zhou et al. [32] studied the particle size distribution of shower-generated aerosols and estimated inhalation doses associated with showering. Their experimental conditions are described above. For hot water, they found that mass median diameters for particles inside the shower stall ranged from about 6.3 *μ*m to about 7.5 *μ*m and that size did not depend on flow rate. Count median diameters ranged from about 4.5 to 5.2 *μ*m. They calculated particle deposition fractions using a model of the human respiratory tract and obtained a total deposition fraction of about 87-88% for mouth breathing and about 90-93% for nose breathing. These values bound the deposition fraction for mixed breathing. The great majority of the particles are deposited in the extrathoracic region, particularly for nose breathing. Only about 7-10% and 1-2%, for mouth and nose breathing, respectively, were estimated to be deposited deep into the lung.

A number of studies have provided information on the particle size distribution of aerosols generated by ultrasonic humidifiers [34, 35, 67, 68]. Rodes et al. [34] reported a count median diameter of 1.8 *μ*m and a mass median diameter of 2.9 *μ*m for wet aerosols from an ultrasonic humidifier using tap water with a total dissolved solids concentration of about 100 mg/L. Wet aerosols from ultrasonic humidifiers dry rapidly at both room humidities and near-saturated conditions; Rodes et al. [34] estimated that less than 0.1 s is required for a 3-*μ*m droplet to dry completely at a room relative humidity of 90%. After drying, Rhodes et al. [34] estimated that the count median diameter for the aerosols from the tap water was about 0.1 *μ*m. Highsmith et al. [35] reported count median diameters of about 0.2—0.3 *μ*m for dry aerosols generated by an ultrasonic humidifier using tap water with a total dissolved solids concentration of about 300 mg/L. Hung et al. [67] (water type not specified) and Umezawa et al. [68] (using tap water) both reported particle size distributions for dry aerosols with peaks at about 0.2 *μ*m. Hung et al. [67] found that the size distributions peaked at about the same aerosol size for both ambient (22°C and 70% relative humidity) and cold-storage (about 5°C and 80-90% relative humidity) conditions. No studies were identified that estimated deposition fractions for inhalation exposures associated with humidifier use.

Bacteria are typically about 0.5 to 5 *μ*m in length. Therefore, at the lower end of the range they can be somewhat larger than the peak sizes of dry aerosols from uncontaminated ultrasonic humidifiers and their sizes extend into the lower part of the range of the sizes of aerosols from hot-water showers. Bacteria generally are smaller than the aerosols associated with showering. Considering their relative sizes, extrathoracic deposition of dry bioaerosols associated with humidifier use is likely to be less than for aerosols generated during showering [69]; it is likely that a larger fraction of bioaerosols generated by humidifier use will be deposited deep in the lung than is the case for aerosols generated during showering. To estimate the dose delivered deep into the lung, the fraction of inhaled particles that reach the alveoli (the end of the respiratory tree in mammals) is needed. This fraction depends on the size distribution of the particles and is generally not known. However, for microbial contaminants associated with bioaerosols the potential dose and the dose deposited in the lungs can be quite different. For example, for *Bacillus anthracis* spores only 22% of an inhaled dose is estimated to be deposited in the lungs for nonhuman primates [70]. (These spores are ellipsoidal in shape with lengths in the range from about 1 to 2 *μ*m and diameters in the range from about 0.5 to 1 *μ*m [71].) Using a model that accounts for transport in the respiratory system, it was estimated that only about 0.08% of such inhaled spores are delivered to the alveoli after 60 min of breathing [72].

Often interest is not in dose per se, but in the response (e.g., infection, death) to the dose. The relationship between dose and response is described by a dose-response model, which is pathogen or chemical specific. For inhalation exposures, the model is generally based on the inhaled (potential) dose. In such cases, the use of an empirical dose-response model will provide estimated responses without the need to estimate applied or absorbed doses. The inhalation doses estimated in this paper are conservative, being potential doses. However, they are generally suited for use with available dose-response functions.

Doses of microorganisms estimated here are based on the implicit assumption that all microorganisms released by showering and humidifier use are viable. However, aerosolization can injure or fragment microorganisms [73]. We did not identify any study that quantifies the damage that aerosolization associated with showering or use of ultrasonic humidifiers causes to microorganisms. Our dose estimates for microorganisms conservatively neglect any loss of viability.

## Summary and Conclusions

This paper presents a detailed approach for assessing the potential system-wide consequences of inhalation exposures that could occur as the result of showering and humidifier use during a contamination event in a water distribution system. The approach utilizes available models for contaminant injection and transport in a WDS, as well as available exposure models for showering and humidifier use. Statistical models for showering frequency and duration are available and were used to describe showering behavior. Given no adequate available model for the times at which showers are taken, we developed timing models for individuals taking one or two showers per day using detailed time-use data collected for the U.S. Because of a lack of behavior models for humidifier use and limitations in the data available for developing these models, we examined the sensitivity of results to assumptions concerning such use. The approach presented here was applied using detailed network models for three actual WDSs.

The approach presented provides generally conservative estimates for doses. In particular, it is assumed that no contaminant losses occur in the WDS and we estimate only potential doses. In addition, any loss of viability of microorganisms during aerosolization of water is neglected.

Both showering and humidifier use could potentially result in significant inhalation doses of contaminants during a contamination event in a WDS. Such activities could also be associated with significant system-wide consequences due to exposures during a contamination event.

Ultrasonic humidifiers and any other types of humidifiers that are similarly efficient at generating aerosols are more important potential sources of inhalation doses of microbial contaminants during contamination events than is showering, which is a much less efficient generator of aerosols. They are also likely to be more important contributors to the system-wide consequences associated with inhalation of microbial contaminants than showering. However, a potential exists for localized adverse effects associated with showering for microbial contaminants with low infectious doses.

Showering could have significant system-wide consequences due to the volatilization of contaminants during a contamination event. The magnitude of inhalation doses associated with showering can be similar to the magnitude of ingestion doses associated with drinking contaminated tap water. The use of humidifiers is unlikely to result in significant exposures to volatile contaminants during contamination events because of the relatively small volume of water used in such units.

The ability to assess the system-wide consequences of humidifier use is hampered by the lack of information needed to develop behavior models for such use. Estimated impacts are sensitive to uncertainties associated with the fraction of the population that uses portable humidifiers, the types of portable humidifiers used, the length of time humidifiers are used each day, and the time when the units are filled. The information needed to develop behavior models for humidifier use should be collected. The work presented here then should be extended by developing and applying behavior models for humidifier use.

The framework presented in this paper provides a flexible approach for assessing the system-wide consequences of inhalation exposures during contamination events. It has been incorporated in publicly available software (TEVA-SPOT) [40] and can be applied to any WDS for which a detailed network model is available.

For cases involving WDSs for which detailed network models are not available, simple bounds were developed that can be used to help assess potential system-wide consequences of inhalation exposures associated with showering and humidifier use.

## Appendix: Bounding Calculations

### Showering

The mass of contaminant that is inhaled by the population during showering (*M*_*Is*_) depends on the mass of contaminant injected into the system (*M*), the fraction of the contaminated water that is used for showering (*f*_*s*_), and the fraction of the contaminant mass that is in the water used in showering that is inhaled (*f*_*Is*_):

$$\begin{array}{c} M_{Is}=f_{Is}f_{s}M \end{array}$$ (A1)

The fraction of the contaminated water that is used for showering (*f*_*s*_) is approximately *v*_*s*_/*V*_*T*_, where *v*_*s*_ is the volume of water used for showering and *V*_*T*_ is the volume of water used for all purposes. The volume of water used for all purposes can be expressed as

$$\begin{array}{c} V_{T}=N_{p}QT_{d} \end{array}$$ (A2)

where *N*_*p*_ is the number of people who use water from the system, *Q* is the per capita withdrawal rate (L/day), and *T*_*d*_ is the time period being considered (in days) when water is contaminated. The volume of water used for showering can be expressed as

$$\begin{array}{c} v_{s}=\sum_{i=1}^{3}p_{i}N_{p}q_{i}T_{d} \end{array}$$ (A3)

where the summation is over the three categories of people grouped by showering frequency (i.e., those who take 0, 1, or 2 showers per day), *p*_*i*_ is the fraction of the population in the *i*th category, and *q*_*i*_ is the daily rate at which water is used by individuals in the *i*th category.

*M*_*Is*_ therefore can be expressed as

$$\begin{array}{c} M_{Is}=\frac{f_{Is}M}{Q}\sum_{i=1}^{3}p_{i}q_{i} \end{array}$$ (A4)

and the maximum number of individuals who can have a dose *d* is given by

$$\begin{array}{c} N_{s}=\frac{M_{Is}}{d}=\frac{f_{Is}M}{Qd}\sum_{i=1}^{3}p_{i}q_{i} \end{array}$$ (A5)

The fraction of individuals in the three showering categories are *p*_1_ = 0.22, *p*_2_ = 0.6, and *p*_3_ = 0.18. (As noted above, we neglect individuals taking three or more showers per day and combine the 1% who do with the 17% who take two showers per day to get 18%.) Also, *q*_1_ = 0 and *q*_3_ = 2*q*_2_. Therefore, $\sum_{i=1}^{3}p_{i}q_{i}=0.96q_{2}$. The daily flow rate for single showers (*q*_2_, in L/day) is equal to the duration of the showers (in min) multiplied by the shower flow rate (in L/min), divided by one day. The average duration of a shower is 7.7 min and the water flow rate is 9 L/min. Therefore, *q*_2_ = 69.3 L/day. Consequently,

$$\begin{array}{c} N_{s}=\frac{66.5f_{Is}M}{Qd} \end{array}$$ (A6)

The fraction of the mass of contaminant that is in the water used for showering that is inhaled, *f*_*Is*_, equals the mass of contaminant inhaled divided by the mass of contaminant in the shower water. The mass of contaminant in the shower water for one shower equals *C*_*w*_ *Q*_*w*_ *t*_1_, where, again, *t*_1_ is the duration of the shower. For volatile contaminants the mass of contaminant inhaled (the potential dose) can be determined using Eq (6). (For contaminants in aerosols, the mass inhaled could be determined using Eq (4).) Using the showering parameters given in Table 2 and *t*_2_ = 2 min, the contaminant mass inhaled during a shower is 1.094*C*_*w*_ mg. Using these same parameters, the mass of contaminant in the shower water for one shower is 69.3*C*_*w*_ mg. Therefore, *f*_*Is*_ = 0.0158 and

$$\begin{array}{c} N_{s}=\frac{1.05M}{Qd} \end{array}$$ (A7)

For the case in which the injection mass is 10 kg and the per capita withdrawal rate is 757 L/day (200 gal/day), the last equation becomes *N* = 13900/*d*, when *d* is in milligrams.

### Humidifier use

Similar to the case for showering, the mass of contaminant that is inhaled by the population during humidifier use (*M*_*Ih*_) depends on the mass of contaminant injected into the system, *M*, the fraction of the contaminated water that is used in humidifiers, *f*_*h*_, and the fraction of the contaminant mass in the water used in humidifiers that is inhaled, *f*_*Ih*_:

$$\begin{array}{c} M_{Ih}=f_{Ih}f_{h}M \end{array}$$ (A8)

The fraction of the contaminated water that is used in humidifiers (*f*_*h*_) is approximately *v*_*h*_/*V*_*T*_, where *v*_*h*_ is the volume of water used in humidifiers. *v*_*h*_ can be expressed as

$$\begin{array}{c} v_{h}=p_{h}N_{p}q_{h}T_{d} \end{array}$$ (A9)

where *p*_*h*_ is the fraction of the population that uses humidifiers and *q*_*h*_ is the daily volume of water used in a humidifier. Therefore, using the expression for *V*_*T*_ from Eq (A2), *M*_*Ih*_ becomes

$$\begin{array}{c} M_{Ih}=\frac{f_{Ih}p_{h}q_{h}M}{Q} \end{array}$$ (A10)

and the maximum number of individuals who can receive a dose *d* is

$$\begin{array}{c} N_{h}=\frac{f_{Ih}p_{h}q_{h}M}{Qd} \end{array}$$ (A11)

The fraction of the contaminant mass in the water used in a humidifier that is inhaled, *f*_*Ih*_, is equal to the mass of contaminant inhaled divided by the mass of contaminant contained in the water used in the humidifier. The mass of contaminant in the humidifier water is simply *C*_*h*_ *S*_*h*_, where *C*_*h*_ is the contaminant concentration in the water used to fill the humidifier and *S*_*h*_ is the volume (size) of the humidifier. The mass of contaminant inhaled is given by Eq (7) for a dose model based on mass-balance approach. For the case in which the dose received from humidifier use is determined by the parameter values given above in the section on estimating inhalation doses from humidifiers, the inhaled dose of contaminant is 0.04*C*_*h*_ mg when the contaminant concentration in the water is *C*_*h*_ mg/L and the volume of water used in the humidifier is 4 L. Consequently,

$$\begin{array}{c} f_{Ih}=\frac{0.04C_{h}}{C_{h}S_{h}}=0.01 \end{array}$$ (A12)

and

$$\begin{array}{c} N_{h}=\frac{0.01p_{h}q_{h}M}{Qd} \end{array}$$ (A13)

If *M* = 10 kg, *q*_*h*_ = 4 L/d, *Q* = 757 L/d (200 gal/d), and *p*_*h*_, the fraction of the population using humidifiers, is 0.2, then *N* = 106/*d*, where *d* is in milligrams.

## Acknowledgments and Disclaimer

This paper has been subjected to EPA’s review and has been approved for publication. Note that approval does not signify that the contents necessarily reflect the views of the Agency. Mention of trade names, products, or services does not convey official EPA approval, endorsement, or recommendation. All post-simulation data analysis and preparation of graphics for this paper were done using R [74]. Kurt Picel provided helpful comments on the manuscript.

Because of the confidentiality of the information, the identities of the WDSs used in this paper and any information that could be used to identify the systems cannot be disclosed.

## Supporting Information

S1 Data EPANET inp Files for Networks E1, E2, and E3. (ZIP)
